# Social, economic, political, and geographical context that counts: meta-review of implementation determinants for policies promoting healthy diet and physical activity

**DOI:** 10.1186/s12889-022-13340-4

**Published:** 2022-05-26

**Authors:** Karolina Lobczowska, Anna Banik, Sarah Forberger, Krzysztof Kaczmarek, Thomas Kubiak, Agnieszka Neumann-Podczaska, Piotr Romaniuk, Marie Scheidmeir, Daniel A. Scheller, Juergen M. Steinacker, Janine Wendt, Marleen P. M. Bekker, Hajo Zeeb, Aleksandra Luszczynska, on behalf of Policy Evaluation Network (PEN) Consortium

**Affiliations:** 1 Department of Psychology in Wroclaw, SWPS University of Social Sciences and Humanities, Ostrowskiego Street 30b, PL53238, Wroclaw, Poland; 2 Leibniz Institute for Prevention Research and Epidemiology – BIPS, Achter Street 30, D28359, Bremen, Germany; 3 Department of Health Policy, School of Health Sciences in Bytom, Medical University of Silesia in Katowice, 18 Piekarska Street, PL41902, Bytom, Poland; 4 Johannes Gutenberg University Mainz, Institute of Psychology, Binger Street 14-16, D55122, Mainz, Germany; 5 Department of Palliative Medicine, Poznan University of Medical Sciences, Russa Street 55, PL61245, Poznan, Poland; 6 Department of Internal Medicine, Division of Sports and Rehabilitation Medicine, University Hospital Ulm, Leimgrubenweg 14; D89075, Ulm, Germany; 7 Wageningen University and Research, Health and Society Group, Center for Space, Place and Society, P.O. Box 8130, bode 60, 6700 EW, Wageningen, The Netherlands; 8 Melbourne Centre for Behavior Change, Melbourne School of Psychological Sciences, University of Melbourne, Redmond Barry Building, Parkville Campus, Melbourne, VIC 3010 Australia

**Keywords:** Policy, Implementation, Diet, Physical activity, Socioeconomic context, Social equity

## Abstract

**Background:**

This meta-review investigated the context-related implementation determinants from seven domains (geographical, epidemiological, sociocultural, economic, ethics-related, political, and legal) that were systematically indicated as occurring during the implementation of obesity prevention policies targeting a healthy diet and a physically active lifestyle.

**Methods:**

Data from nine databases and documentation of nine major stakeholders were searched for the purpose of this preregistered meta-review (#CRD42019133341). Context-related determinants were considered strongly supported if they were indicated in ≥60% of the reviews/stakeholder documents. The ROBIS tool and the Methodological Quality Checklist-SP were used to assess the quality-related risk of bias.

**Results:**

Published reviews (*k* = 25) and stakeholder documents that reviewed the evidence of policy implementation (*k* = 17) were included. Across documents, the following six determinants from three context domains received strong support: economic resources at the macro (66.7% of analyzed documents) and meso/micro levels (71.4%); sociocultural context determinants at the meso/micro level, references to knowledge/beliefs/abilities of target groups (69.0%) and implementers (73.8%); political context determinants (interrelated policies supported in 71.4% of analyzed reviews/documents; policies within organizations, 69.0%).

**Conclusions:**

These findings indicate that sociocultural, economic, and political contexts need to be accounted for when formulating plans for the implementation of a healthy diet and physical activity/sedentary behavior policies.

**Supplementary Information:**

The online version contains supplementary material available at 10.1186/s12889-022-13340-4.

## Background

Obesity rates and the global burden of diseases attributable to poor diet, low physical activity (PA), and high sedentary behavior (SB) have been increasing during the last two decades [1, 2]. International organizations responsible for setting health policy standards have consistently emphasized that any public health policy should be developed and implemented to promote better health for everyone [3, 4]. Therefore, health policies aimed at preventing non-communicable diseases through a healthy diet and PA might be envisaged as tools that reach various populations that differ in social and economic situations [3, 4]. To achieve this ambitious goal, policy implementation processes should account for social, cultural, economic, and political contexts [4]. This meta-review aims to summarize the evidence on the context-related determinants that occur during the implementation process of obesity-prevention policies targeting a healthy diet and PA/SB.

Policies are defined as actions developed and implemented to achieve specific goals within a society, with national or regional governments taking part in the development and/or implementation of these actions [5, 6]. In contrast, interventions are actions targeting similar goals but not yet endorsed, enabled, or executed by regional or national governments [6]. Policy implementation is the process of putting to use or integrating a policy within target settings (or systems) [7].

Policy implementation frameworks, such as the consolidated framework for implementation research (CFIR) [8], list implementation determinants that refer to the characteristics of organizations, communities, and broader policy systems. The CFIR-based meta-reviews indicated that crucial implementation determinants for diet and PA/SB policies include implementation costs, networking with other organizations/communities, external policies, structural characteristics of the setting, implementation climate, and readiness for implementation [9]. Implementation of policies promoting a healthy diet, PA increase, or SB reduction has been recognized as a process that operates in a multidimensional context [3, 10–12]. Thus, in addition to determinants accounted for in the CFIR framework, contextual factors addressing health inequalities (socioeconomic determinants, culture, geographic isolation) might also play a role in policy implementation [3, 10, 12] and help clarify why the implementation of a healthy diet or PA-promoting policy is successful in one community but not in others [13].

According to the context and implementation of a complex intervention framework (CICI), context factors might be represented at the macro (e.g., country-level characteristics), meso, and micro levels (e.g., characteristics of the target organizations, target families, or target individuals) [10, 12]. The CICI framework proposes seven context domains [10]. The geographical context refers to the broader physical environment, such as the built environment in a local community that hinders physical activity (the meso/micro level). The epidemiological context deals with the demographic structure and distribution of diseases in a target population (the macro level) and captures micro-level determinants, such as the needs of the target population (determined by epidemiology but also psychosocial or physical needs). The sociocultural context comprises core ideas and values essential for the culture of the target group (e.g., members of specific ethnic groups) and meso/micro level factors, such as values, beliefs, and knowledge of the target individuals and of those who enforce or deliver the implementation (implementation actors). The economic context consists of economic resources at the macro level (e.g., national funds for specific actions) and meso/micro level factors, such as access to the economic resources of individuals or organizations. The ethical context addresses norms and rules that reflect moral positions and determine the standards of conduct of individuals or institutions (the meso/micro level) or the population (the macro level), such as guidelines referring to consent or stigma issues. The political context addresses interactions of existing national policies (the macro level) with the newly implemented policies, policies that shape actions within and across relevant sectors (e.g., health and education), and formal and informal policies, interests, and pressure groups that govern organizational and individual actions (the micro level). Finally, the legal context refers to the existing rules and codified regulations established to govern societal actions and interests [10].

Some similarities exist in the processes of implementing different policies promoting a healthy diet and PA because some of them operate within similar environments (e.g., a local community) and have the common goal of reducing obesity and obesity-related non-communicable diseases [14]. Thus, the implementation of some policies might have common context-related implementation determinants. In contrast, some context-related determinants are likely to occur during the implementation of policies that target a specific behavior (e.g., healthy diet vs. PA) in a specific setting [11, 12, 15, 16]. The literature also suggests that the implementation of policies developed for specific target groups, such as populations at risk for obesity, might depend on specific contextual determinants, such as healthcare system characteristics [17].

Several systematic reviews analyzing determinants for healthy diet and PA policies [11, 15, 18–20] provide insights into specific categories of implementation determinants. For example, using the CFIR framework, Lobczowska et al. [9] elicited determinants that are closely related to the characteristics of the specific policy (e.g., its complexity or quality), characteristics of the networks and organizations in which the policy is implemented (e.g., implementation climate within the organizations involved), characteristics of the individuals involved in the implementation (e.g., referring to the identification with an organization), and implementation process characteristics (e.g., referring to planning and evaluating implementation). The CFIR-based approach [8, 9] narrows down the implementation determinants to those that are proximal to the implementation of specific policy. The CFIR misses a broader political, legal, and ethical context, in which the implementation takes place [8, 9]. In particular, the CFIR-based approach [8, 9] does not provide an insight into the economic, education-related, demographic, geographical, and cultural factors, that are the key indicators of social inequalities [3, 4] and as such should be considered in health policy research [4]. Determinants representing a broader context, related to social inequalities, were not systematically considered in existing reviews on healthy diet and PA policy implementation processes [11, 15, 18–20], although the issue of reducing health inequalities across various populations remains a key task of these policies [4]. Furthermore, there is no overarching synthesis of research on these broader context-related determinants that occur in the implementation of dietary PA/SB policies targeting specific subpopulations in specific settings (e.g., children/adolescents at school, employees at work, and populations at risk for obesity in clinical/education/social services settings).

Recent research on policy implementation highlighted the need for a more thorough investigation of the stakeholders’ position, in order to obtain a fuller picture of implementation processes and to increase the potential impact of research on future policy directions [21]. Using accumulating evidence, major international and national stakeholders are issuing documents on developing, implementing, and evaluating a healthy diet and PA/SB policies (e.g., the World Health Organization [16]). These documents were developed to guide governments in the formation and implementation of regional and national policies [16]. The synthesis of stakeholder documents might help identify similarities/differences between empirical evidence (accumulating in reviews) and policy-guiding stakeholder documents. Documents of major stakeholders that discuss implementation processes are based on empirical evidence, but they are also shaped by the stakeholders’ political intentions, agendas, and interests, and the influence or resources the contributing parties bring to shape the development of respective documents [21]. Major stakeholder documents may be influenced by organizations/individuals who are actively involved in the policy implementation processes, and whose experience in practice may complement the results of published empirical evidence [21]. In sum, the stakeholder documents may capture the influences operating within existing complex policy systems, practice-based solutions and insights, combined with empirical evidence. Thus, stakeholder documents are considered to represent grey literature relevant in an investigation of policy implementation [22], and as such, they may be included to complement the findings obtained in systematic reviews. It is unclear, however, whether published reviews differ in their findings on implementation determinants relative to the position of major stakeholders, guiding the decisions of policymakers and practitioners.

### Aims

The purpose of this meta-review was to synthesize the evidence (accumulated in reviews and evidence-based stakeholder documents) for the occurrence of context-related implementation determinants of policies targeting PA/SB or a healthy diet in the general population, PA/SB or healthy diet policies targeting specific settings (school, workplace), and PA/SB or healthy diet policies targeting populations at risk for obesity. In contrast to previous research focusing on implementation determinants closely related to the characteristics of policy itself, involved organizations, and processes of implementation planning or evaluation [9] we investigated an occurrence of a different type of policy implementation determinants, reflecting a broader sociocultural context and processes contributing to social inequalities.

In particular, using the CICI framework [10] we aimed to investigate: (1) the context-related implementation determinants (in the following domains: geographical, epidemiological, sociocultural, economic, political, ethical, legal) that occurred during the implementation process of policies targeting a healthy diet and PA/SB reported in reviews/stakeholder documents; (2) the differences and similarities in corroboration with context-related determinants of policy implementation obtained in: (a) reviews versus stakeholder documents, (b) reviews/stakeholder documents addressing healthy diet policies versus PA/SB policies, and (c) reviews/stakeholder documents addressing PA/SB or healthy diet policies targeting specific populations: children/adolescents in school setting versus employees in workplace settings versus children/adolescents/adults at risk for obesity (in clinical, education, or social services settings).

## Method

### Materials and general procedures

A meta-review (systematic review of reviews [23]) integrating empirical evidence from existing systematic, realist, scoping reviews, and stakeholder documents was conducted. This study was conducted in line with the Preferred Reporting Items for Systematic Reviews and Meta-Analyses (PRISMA) guidelines [24, 25] and following best-practice recommendations for meta-reviews [23]. The present study reports findings obtained in a search conducted in a larger systematic review (registered with the PROSPERO database; no. CRD42019133341) aimed at eliciting various implementation determinants for healthy diet and PA/SB policies.

This meta-review was based on data obtained in records identified through database searching conducted by Lobczowska et al. [9]. The use of the same records was possible because the keywords used in the search by Lobczowska et al. [9] referred to any determinants of implementation of healthy diet and PA/SB policies, and thus allowed to reach the objectives of the present review, focusing on the broader context-specific determinants. However, data screening, data coding, and data analyses were conducted independently in both reviews, to address their distinct goals, referring to different types of implementation determinants.

### Published reviews: search strategy, inclusion, and exclusion criteria

**Fig. 1:**
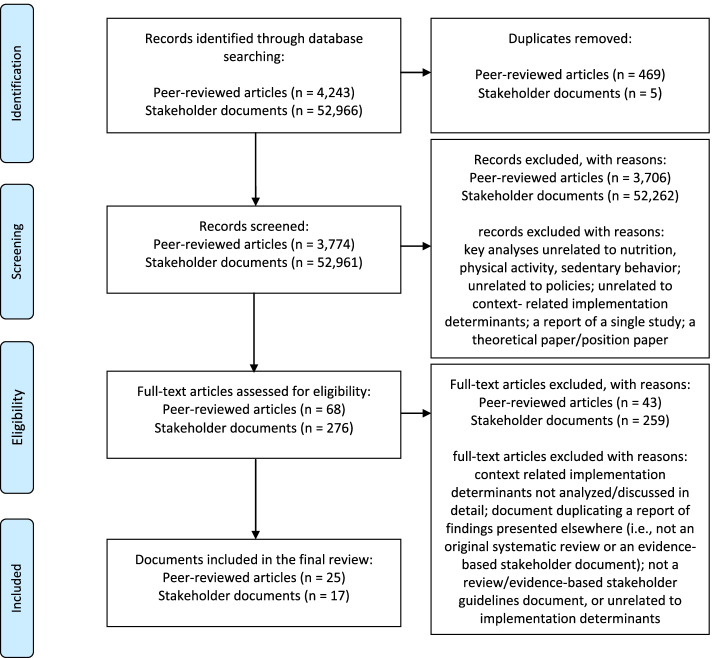
The flow chart: selection processes for peer-reviewed articles and stakeholder documents

The following databases were searched: PsycINFO, PsycARTICLES, Health Source: Nursing/Academic Edition, MEDLINE, Academic Search Ultimate, AGRICOLA (all six databases accessed via EBSCO Host), the Cochrane Database of Systematic Reviews (accessed via Cochrane Library), the Database of Abstracts of Reviews of Effects (accessed via the University of York Centre for Reviews and Dissemination), and Scopus. As suggested by Hennessy et al. [23], utilizing as many relevant databases as possible is advised, not only to ensure that relevant reviews were retrieved but also to reduce a potential selection bias. Our robust approach resulted in an overlap of databases/entries, but further stages of search procedures (see Fig. 1) accounted for the removal of duplicates. Documents published between the inception of the databases and February 2020 were included. Additionally, reference lists of reviews were manually searched, and keyword-based searches of implementation journals (e.g., Health Research Policy and Systems, Policy Studies) were performed.

The search applied a string with five groups of keywords that referred to: (1) implementation; (2) barriers and facilitators (barrier* OR facilitat* OR determinant* OR factor* OR affect*, etc.; 10 keywords); (3) the type of action (i.e., policy); (4) the outcomes (“physical activity” OR active OR exercise OR sedentary OR sport, etc.; 23 keywords); and (5) review (“data synthesis” OR “synthesis of data” OR “descriptive synthesis” OR “evidence synthesis” OR “synthesis of evidence” OR “synthesis of available evidence,” etc.; 124 keywords, recommended in the guidelines for the use of keywords to identify systematic reviews when conducting a meta-review [23]). The full list of keywords is included in Additional file 1 (Supplementary Table S1). The keywords were selected based on previous reviews addressing related issues [18, 19, 26, 27].

In case of this meta-review the chosen strategy was to use a broad, inclusive search string (e.g., applying multiple terms that could represent the investigated processes; using only basic operators [AND, OR], and applying no specific limits) that could be used across the databases. The feasibility of the string was pretested across the databases, before the search was initiated. The decision of using the broad search string increased the number of identified entries, but reduced the likelihood of excluding relevant documents during the first stages of the search process. Figure 1 presents the details of the data selection process. A preliminary search yielded *k* = 4243 records. All identified abstracts were screened by two researchers (KL and AB). Any conflicts related to the potential inclusion of a document were resolved through discussions with a third researcher (AL).

The following inclusion criteria were applied: quantitative and qualitative reviews (designs including systematic, scoping, and realist reviews) of original research, providing empirical evidence on implementation determinants for policies promoting a healthy diet, PA promotion policies, or PA promotion/SB reduction policies published in peer-reviewed English-language journals. The following types of documents were excluded: original studies (i.e., research that did not aim at providing a review but focused on reporting new results of an original study), dissertations, protocols, conference materials, and book chapters; reviews that did not provide any empirical evidence for the role of implementation determinants as predictors of the implementation process or policy effectiveness indicators, reviews of policy guidelines (not original research), and reviews of theoretical frameworks.

### Stakeholder documents: search strategy, inclusion and exclusion criteria

We included stakeholders representing governmental and non-governmental organizations issuing evidence-based policy guidelines (in English) for diet, PA, and/or SB policies at the national or international level. The inclusion of stakeholder documents allows us to cover the grey literature [22], and is consistent with the approach applied in previous reviews on implementation determinants [27]. Publicly available stakeholder websites (e.g., repositories of strategy documents, policy guidelines, and best practice guidelines) were searched to identify potentially relevant documents that addressed determinants of the implementation of healthy diet policies, PA promotion policies, or SB reduction policies, and included a review of evidence on policy implementation determinants. The stakeholders were: the National Institute for Health and Care Excellence (United Kingdom), the European Commission (e.g., Consumers, Health, Agriculture and Food Executive Agency), World Health Organization, Regional Office for Europe, Centers for Disease Control and Prevention (USA), National Academy of Medicine (USA), Australian Department of Health, National Health and Medical Research Council (Australia), Organization for Economic Co-operation and Development, and Food and Agriculture Organization of the United Nations. For a similar strategy of identifying and selecting stakeholders, see prior research [26, 27]. The databases were searched from their inception until February 2020 using the same combination of five groups of keywords, as applied in the search for reviews. No search filters were used in this study.

Based on prior research [18, 19, 26, 27], the inclusion criteria were as follows: documents issued in English; non-systematic position reviews published by a stakeholder and stakeholder documents focusing on reviewing evidence-based implementation determinants of policies targeting a healthy diet, PA, and/or SB; using research evidence to discuss the implementation process and its determinants (i.e., including references to original research or reviews of original research when indicating the importance of a context-related determinant); and documents developed and officially endorsed by a respective stakeholder. The exclusion criteria were the same as those applied to the published reviews.

The initial search identified 52,966 potentially relevant documents (see Fig. 1). The documents were screened, and the respective data were coded by at least two researchers (PR, KK, ANP, MS, TK, JW, DAS, KL, or AL).

### Data extraction

All stages of data extraction, selection, and coding were conducted by at least two researchers. Any disagreements during the data extraction process were resolved by a consensus method (searching for possible rating errors, followed by discussion and arbitration by a third researcher [28]).

Descriptive data (see Supplementary Table S1, Additional file 2) and data necessary for quality evaluations were extracted by two researchers (KL and AL) and verified by a third researcher (AB). Extracted data included: (1) the descriptive characteristics of the included reviews/stakeholder documents (e.g., number, design, and objectives of original studies included in the review, a framework used to guide and organize the review findings, target population and settings, analyzed behavior); (2) data concerning determinants (definitions of implementation determinants if provided by authors of reviews/stakeholder documents; a list of determinants of implementation for which the explicit reference for a significant role/importance of a respective determinant was reported in the results sections of the included reviews; evidence-based determinants indicated as relevant in the stakeholder documents); and (3) data necessary for a quality evaluation and the assessment of the risk of bias.

The potential context-related determinants for the implementation of policies were extracted from each document (Supplementary Table S1, Additional File 2). In particular, the names of the implementation determinants (as documented by the authors of the original review/stakeholder document), their operationalization, and/or definitions were retrieved. The determinants for the implementation of policies were extracted only if they were discussed in the results section of the reviews or, in the case of stakeholder documents, supported by empirical evidence (as indicated by the references provided).

### Data coding

Reviews and stakeholder documents were coded as referring to:

- policy, if any of the original studies included in the respective review/document addressed actions aimed at promoting a healthy diet and active lifestyle that were developed and implemented (or enforced) with local authorities or national government participating in respective processes [5, 6]. In contrast to policies, interventions are actions developed and implemented without the participation of local authorities or the national government, although such actions might address similar aims [5, 6];
- context-related policy implementation determinants, if the review/stakeholder document addressed determinants from the seven context domains included in the CICI framework [10] (i.e., geographical, epidemiological, sociocultural, economic, ethics-related, political, and legal domains);
- healthy diet, if the review/stakeholder document addressed policies for food composition, food labeling, healthy nutrition promotion, food provision, food retail, food prices, or food trade and investment [29];
- PA or SB, if reviews/stakeholder documents targeted behaviors across sectors such as healthcare, sport/recreation, education, transport, environment, urban design, urban planning, etc. [30]. Reviews/stakeholder documents addressing multiple behaviors were grouped into documents addressing: (1) policies aimed at a healthy diet, PA increase, and/or SB reduction; and (2) policies aimed at a healthy diet, PA increase, SB reduction, and other behaviors (e.g., tobacco use and alcohol use);
- children and adolescents in school settings if the review/stakeholder document referred to a healthy diet or PA/SB policies targeting children/adolescents in an education-related setting, including preschools, education daycare centers for young children, primary, secondary, and high schools;
- employees in the workplace setting, if the review/stakeholder document referred to healthy diet or PA/SB policies targeting populations of employees or managers in workplace settings;
- populations of children, adolescents, or adults at risk for obesity (in various clinical and non-clinical settings), if the review/stakeholder document discussed healthy diet or PA/SB policies developed for a specific target population, such as pregnant or postpartum women at risk for weight gain, people with diabetes at risk for (further) weight gain, or children and adolescents with overweight/obesity; these policies were mostly implemented in clinical, education or social service settings.

Context-related policy implementation determinants were allocated into seven domains of the CICI framework using the original description of domains [10]. All determinants were also coded as belonging to the macro level (national or country level) and meso/micro level (community/organizational or individual level), in line with the definitions provided by Pfadenhauer et al. [10] and Swinburn et al. [12]. Sixteen groups of context-related determinants were developed: (1) geographical (*k* = 2 groups of context-related determinants), including the broader physical environment, such as geographical isolation (the macro level); infrastructure in the setting (the meso/micro level); (2) epidemiological (*k* = 2), including the distribution of diseases, disease burden, demographics - age, gender (the macro level), and physical and/or psychological needs of target groups (the meso/micro level); (3) sociocultural (*k* = 3), including culture-related ideas, symbols, roles, and values (the macro level); target groups’ knowledge, beliefs, abilities (the meso/micro level); implementers’ knowledge, beliefs, abilities (the meso/micro level); (4) economic (*k* = 2), including the country’s economic resources (the macro level); individuals’ or organizations’ economic resources (the meso/micro level); (5) ethical (*k* = 3), including standards of conduct, ethical principles at the national (macro) level; target individuals’ ethics-related beliefs and principles (the meso/micro level); implementers’ ethics-related beliefs and principles (the meso/micro level); (6) political (*k* = 3), including interrelated policies, political pressures operating at the macro level; sectorial policies and system properties in health care, education, food production, and retail sectors (the macro level); policies in organizations (the meso/micro level); and (7) legal (*k* = 1), including enforced laws, rules/regulations established to protect population rights, and societal interests (the macro level). Additional File 1 (Supplementary Table S2) presents further coding details for the CICI-based context-related determinants.

### Quality assessment

Two researchers (KL and AB) independently rated the included reviews and stakeholder documents. Reviews were assessed for quality using criteria from the ROBIS tool to evaluate the risk of bias in systematic reviews [31]. The risk of bias in stakeholder documents was assessed using the Methodological Quality Checklist for Stakeholder Documents and Position Papers (MQC-SP [26, 27];). Thresholds for low, moderate, and high risk of bias were defined in line with the rules indicated in the respective assessment tools [26, 27, 31]. The obtained scores are reported in Additional file 2 (Supplementary Table S1). The values of the concordance coefficients (intra-class correlation) for quality assessment ranged from 0.71 to 0.90 (all *p*s < .003).

### Data analysis and synthesis

Reviews and stakeholder documents were coded as not corroborating (−) or providing corroboration (+) for the occurrence of the context-related determinant in the policy implementation process (Additional file 1, Supplementary Tables S3 and S4). The reviews of the quantitative studies were coded to corroborate the presence of a context-related determinant if the results section of the review indicated that: (1) the respective determinant was identified in the review as significantly associated with another characteristic of the implementation processes or their outcomes (e.g., policy adoption); and (2) the determinant was identified in the review as occurring during the implementation process (e.g., an indication existed of the level of intensity/frequency or median/range values of the determinant in the results section of a review). The included reviews used various thresholds to identify the occurrence of a determinant (e.g., mean, range, and occurrence in the analyzed data). Therefore, we coded the determinant as “indicated in the review” if the review’s results concluded that the determinant was present in the implementation process. The reviews of the qualitative studies were coded as corroborating the context-related determinant if the results section of the review indicated that the respective determinant was identified in the original qualitative data discussed in the review. Stakeholder documents were coded as providing corroboration for the presence of the context-related determinant in the implementation process if the section of the document providing guidelines/best practices overviews listed a determinant, indicated its significance/importance/need for consideration in policy implementation processes, and provided a reference to the empirical evidence backing a respective statement.

Implementation determinants indicated in ≥50% of reviews/stakeholder documents were preliminarily supported by the analyzed data. The determinants indicated in ≥60% of the analyzed reviews/stakeholder documents were strongly supported by the analyzed data. The thresholds did not account for the number or quality of the original studies included in the respective review/stakeholder document. Similar thresholds were applied in previous meta-reviews in the context of policies and interventions promoting a healthy diet and PA [26, 27, 32, 33].

Reviews versus stakeholder document comparisons of healthy nutrition and PA/SB policies were conducted. Context-related determinants that obtained strong support were listed, and similarities and differences in the lists were identified. The implementation determinants for diet versus PA/SB policies were compared using reviews/stakeholder documents that addressed only the implementation of policies targeting the respective behavior (i.e., nutrition policies only vs. PA/SB policies only; reviews/stakeholder documents combining multiple behavior policies were excluded). Data on healthy diet and PA/SB policies targeting specific populations in specific settings (children/adolescents at school, employees in the workplace, and populations at risk for obesity in clinical/education/social services settings) were summarized separately, listing the implementation determinants that obtained strong support.

## Results

A total of *k* = 25 reviews [15, 18, 19, 34–54] and *k* = 17 stakeholder documents [17, 55–70] were included. The reviews reported findings from 747 original studies. Additional file 1 (Supplementary Table S5) presents the details of the populations analyzed, policy target behaviors, and settings. Supplementary Table S6 (Additional file 1) lists examples of the context-related determinants reported in the respective reviews/stakeholder documents (for a full list of determinants, see Additional file 2).

Across the reviews and stakeholder documents, *k* = 12 focused on the implementation of healthy diet policies targeting various populations/settings, *k* = 9 addressed the implementation of PA/SB policies in various populations/settings, and *k* = 6 (reviews only) addressed the implementation of PA and diet policies in various populations/settings (see Supplementary Table S7, Additional file 1). The remaining reviews/stakeholder documents (*k* = 15) discussed the implementation of a combination of diet, PA, and SB policies.

Reviews/stakeholder documents addressing the implementation of policies among children/adolescents in school settings, adult employees in the workplace, and people at risk of obesity in clinical/education/social services settings were heterogeneous regarding the target policy behavior (children/adolescents in school: 3 reviews/stakeholder documents addressing diet, 1 - PA/SB, 3 - PA, 3 - diet/PA/SB; employees in the workplace: 1 review/stakeholder document addressing PA; 4 - diet/PA/SB; populations at risk for obesity: 1 addressing diet, 5 - addressing diet/PA/SB) (see Supplementary Table 7, Additional file 1). Strong heterogeneity and small numbers of reviews/stakeholder documents addressing the respective populations/settings (e.g., employees in the workplace) did not allow for comparisons of the determinants of implementation of diet versus PA/SB policies within a target population/setting. Additionally, a subgroup analysis of context-related determinants in populations at risk for obesity was not possible because each review/stakeholder document addressed a different target subpopulation (e.g., one referred to people at risk for diabetes, another addressed pregnant and postpartum women).

Across 25 reviews, only 3 [18, 19, 54] reported quantitative results that indicated associations between a determinant and any other implementation process-related variable. Only one meta-analysis was conducted; results of this meta-analysis showed no significant effects of the determinants of the implementation outcome variables based on three original studies [58]. The majority of the reviews (72.0%, 18 out of 25) provided a narrative synthesis of the results, in which a context-related determinant identified in the included data was indicated, followed by examples of original research that reported the respective determinants. Only 7 out of 25 reviews (28.0%) [14, 17, 42, 50, 52, 53, 58] provided some descriptive statistics, clarifying a proportion of studies that indicated the occurrence of a respective determinant, compared with the total number of relevant original studies.

The risk of bias scores obtained using ROBIS [30] and MCQ-SP [26, 27] are reported in Additional file 2 (Supplementary Table S1). Across the reviews, 48% (*k* = 12) were evaluated as representing a low risk of bias across 5 criteria of ROBIS [31], 24% (*k* = 6) were considered to represent low risk across 4 criteria, and 8% (*k* = 2) had low risk in 3 criteria. The remaining 20% (*k* = 5) of the reviews were evaluated as having high or unclear risk in ≥3 criteria. Regarding stakeholder documents, 47% (*k* = 8) were evaluated as having a low risk of bias (high quality in MQC-SP tool [26, 27]), 29% (*k* = 5) had moderate quality/risk of bias, and 24% had high risk/low quality (*k* = 4).

### Overall support for context-related policy implementation determinants

Across all *k* = 42 reviews/stakeholder documents, 6 implementation determinants from 3 context domains received strong support, with 2 referring to the macro-level and 4 to the meso/micro-level (Table 1). They comprised: economic resources at the macro level (66.7% of *k* = 42 reviews/stakeholder documents), economic resources at the meso/micro level (71.4%), and sociocultural context at the meso/micro level, referring to the beliefs, knowledge, and capabilities of the target group (69.0%) and implementers (73.8%); political context determinants at the macro level (interrelated policies; 71.4%) and the meso/micro level (policies within organizations; 69.0%). Preliminary support (52.4%) was also obtained for the geographical context-related determinants (the meso/micro level), sociocultural context-related determinants (culture-related ideas, roles, and values at the macro level; 54.8%), and political context determinants (concerning sectorial policies at the macro level; 57.1%). The ethics domain was the least supported, with 7.1% of *k* = 42 reviews/stakeholder documents providing some support for ethics-related standards or norms.

**Table 1:** Evidence from reviews and stakeholder documents supporting the occurrence of context-related implementation determinants for policies promoting a healthy diet and physical activity

Context domains Context-related determinants based on CICI framework	Total (*k* = 42) %	Reviews only (*k* = 25) %	Stakeholder documents only (*k* = 17) %	Diet (*k* = 12) %	PA/SB (*k* = 9) %	Children in schools (*k* = 10) %	Employees in the workplace (*k* = 5) %	Populations at-risk for obesity in clinical/education settings (*k* = 6) %
**Geographical**								
Broader physical environment (macro level)^*^	42.9	36.0	*52.9*	41.7	44.4	20.0	**60.0**	*50.0*
Infrastructure in the setting (meso/micro level)	*52.4*	*56.0*	47.1	33.3	**66.7**	**80.0**	**60.0**	*50.0*
**Epidemiological**								
Disease distribution, disease burden, demographics (macro level)	31.0	8.0	**64.7**	41.7	0	0	0	**83.3**
Target group needs (meso/micro level)	38.1	32.0	47.1	33.3	11.1	30.0	20.0	**66.7**
**Sociocultural**								
Culture-related ideas, symbols, roles, values (macro level)	*54.8*	40.0	**76.5**	*58.3*	22.2	40.0	20.0	**100**
Target group: knowledge, beliefs, abilities (meso/micro level)	**69.0**	**72.0**	**64.7**	**75.0**	**88.9**	**60.0**	**60.0**	**83.3**
Implementers: knowledge, beliefs, abilities (meso/micro level)	**73.8**	**76.0**	**70.6**	**75.0**	**77.8**	**90.0**	20.0	**100**
**Economic**								
Economic resources of communities (macro level)	**66.7**	*56.0*	**82.4**	**91.7**	33.3	*50.0*	0	**100**
Individual/organizational economic resources (meso/micro level)	**71.4**	**68.0**	**76.5**	**75.0**	*55.6*	**80.0**	**80.0**	**83.3**
**Ethics**								
Standards of conduct, ethical norms, stigma (macro level)	7.1	0	17.6	0	0	0	0	16.7
Target group: standards, norms, stigma (meso/micro level)	0	0	0	0	0	0	0	0
Implementers: standards, norms, stigma (meso/micro level)	0	0	0	0	0	0	0	0
**Political**								
Interrelated policies, political pressure (macro level)	**71.4**	**64.0**	**82.4**	**91.7**	*55.6*	**70.0**	0	**100**
Policies in organizations involved/partner organizations (meso/micro level)	**69.0**	**64.0**	**76.5**	**83.3**	*55.6*	**60.0**	0	**83.3**
Sectorial policies: health care, education, food production and retail system properties (macro level)	*57.1*	48.0	**70.6**	**75.0**	33.3	30.0	0	**100**
**Legal**								
Rules/regulations established to protect population rights/societal interests, enforced laws (macro level)	45.2	36.0	*58.8*	**75.0**	11.1	20.0	0	**66.7**

*Note*: *PA* physical activity; *SB* sedentary behavior; % - the percentage or the reviews/stakeholder documents that provided an explicit reference for a significant role/importance of a respective context-related implementation determinant; Total - reviews/stakeholder documents addressing implementation determinants for healthy diet and PA/SB policies; Reviews - reviews addressing implementation determinants for healthy diet and PA/SB policies; Stakeholder - stakeholder documents addressing implementation determinants for healthy diet or PA/SB policies; Diet - reviews/stakeholder documents addressing implementation of healthy diet policies across various populations/settings; PA/SB - reviews/stakeholder documents addressing implementation of PA/SB policies across various populations/settings; Children and schools - reviews/stakeholder documents addressing implementation of healthy diet or PA/SB polices targeting children/adolescents in school settings; Employees at workplaces - reviews/stakeholder documents addressing implementation of healthy diet or PA/SB polices targeting employees in workplace settings; Populations at risk for obesity in clinical/education settings - reviews/stakeholder documents addressing the implementation of healthy diet or PA/SB polices targeting populations at risk for obesity in clinical, educational, or social services settings

^*^ - Context-related implementation determinants might be divided into macro (nationwide), meso (organizational)/micro (individual) levels. The percentage of implementation determinants corroborated in ≥50% of reviews/stakeholder documents (preliminarily supported) are marked in italics. Percentage of implementation determinants corroborated in ≥60% of analyzed reviews/stakeholders (considered strongly supported) are marked in bold font

### Context-related implementation determinants supported in reviews vs. supported in stakeholder documents

When the findings obtained solely in reviews (*k* = 25) were considered, 5 context-related determinants were strongly supported (64.0–72.0% of *k* = 42 reviews), including: sociocultural at the meso/micro level, referring to knowledge/beliefs/abilities of the target population and of the implementers; economic at the meso/micro level, referring to individual/organizational resources; and political (the macro and meso/micro level), referring to interrelated policies and policies in the involved/partner organizations (Table 1).

The same 5 context determinants were strongly supported (64.7–82.4%) in *k* = 17 stakeholder documents. Additionally, stakeholder documents provided strong support (64.7–82.4% of *k* = 17 stakeholder documents) for 4 context-related macro-level determinants, including culture, economic resources, sectorial system policies, and interrelated policies/political pressure. Finally, only stakeholder documents provided any corroboration of context-related determinants from the ethics domain (at the macro level). Support for this domain was limited (17.6%) and found only in documents addressing policies targeting populations at risk for obesity.

### Context-related implementation determinants in healthy diet policies vs. PA and PA/SB policies

Eight context-related implementation determinants from 4 context domains were strongly supported when the implementation of healthy diet policies (*k* = 12) was considered (Table 1). They included: 2 sociocultural meso/micro-level context-related determinants, referring to the knowledge/beliefs/abilities of the target population and implementers (both supported in 75% of *k* = 12 reviews/stakeholder documents referring to healthy diet policies); economic determinants at the macro (91.7%) and the meso/micro (75.0%) levels; political determinants at the macro (75.0–91.7%) and meso/micro (83.3%) levels; and legal context-related determinants operating at the macro level (75.0%).

Only 3 context-related determinants received strong support from reviews and stakeholder documents on the implementation of PA/SB policies (*k* = 9). They addressed the meso/micro-level context in the geographical domain (setting’s infrastructure, 66.7%) and the sociocultural domain, referring to the knowledge/beliefs/abilities of the target population (88.9%) and implementers (77.8%).

### Differences in context-related implementation determinants between healthy diet/PA/SB policies for children and adolescents in school settings, employees in workplace settings, and populations at risk for obesity in clinical/education/social services settings

The analysis of the corroboration of context-related implementation determinants showed more differences than similarities across healthy diet, PA promotion, or PA/SB policies designed for children and adolescents in school, employees in the workplace, and populations at risk for obesity (Table 1). For policies targeting children/adolescents in school settings, 6 context-related determinants (including 2 at the macro level) were strongly supported. Only 4 (including 1 at the macro level) determinants were strongly supported for employees in the workplace, whereas 11 context-related determinants (including 6 at the macro level) were strongly supported in reviews/stakeholder documents on policies targeting populations at risk for obesity.

Similarities and differences were found in the level of support for context-related determinants that were identified for the implementation of policies targeting children/adolescents in school settings, (2) employees in the workplace, and (3) people at risk for obesity in healthcare/educational/social services setting. In the case of school setting-related policies targeting children/adolescents (*k* = 10), context-related determinants from the geographical, sociocultural, economic, and political domains were strongly supported (60.0–90.0%). In the case of workplace setting policies (*k* = 5) targeting employees, the geographical, sociocultural, and economic context domains were also strongly supported (60.0–80.0%), but the political domain did not receive strong support. In the case of the implementation of policies targeting populations at risk for obesity (*k* = 6), the relevance of the geographical domain wasn't strongly supported, whereas the following three domains received strong support: sociocultural, economic, and political (83.3–100%). Additionally, context-related determinants from epidemiological and legal context domains were strongly supported (66.7–83.3%) in reviews/stakeholder documents on policies targeting populations at risk for obesity.

### Summary of findings across seven context domains

**Fig. 2:**
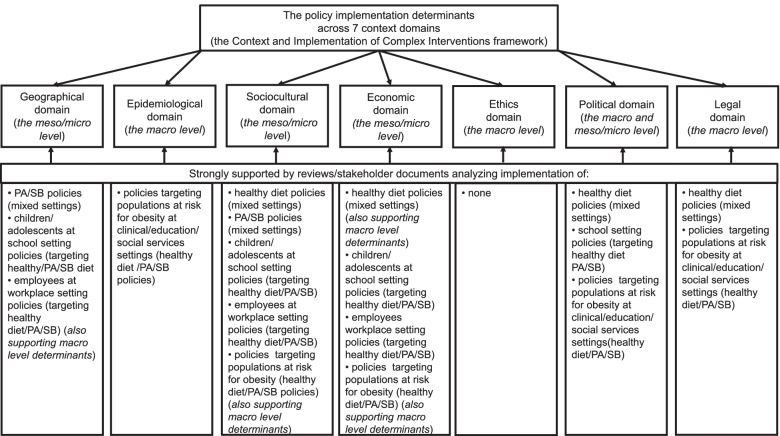
Strong support obtained in *k* = 42 reviews and stakeholder documents addressing policy implementation determinants. *Note*: Context-related implementation determinants corroborated in ≥60% of analyzed reviews/stakeholder documents were considered as strongly supported by analyzed data. Context-related determinants might be represented at the macro (e.g., country-level characteristics), meso (e.g., organization-level characteristics), and micro levels (e.g., characteristics of target families or target individuals)

Figure 2 summarizes the strong support obtained for the seven context domains of the CICI framework. The support could be obtained across reviews/stakeholder documents addressing the implementation of the following types of policies: targeting a healthy diet across various populations (*k* = 12 reviews/stakeholder documents), targeting PA/SB across various populations (*k* = 9), healthy diet or PA/SB policies for children/adolescents in school settings (*k* = 10); healthy diet or PA/SB policies for adult employees in workplace settings (*k* = 5); and healthy diet or PA/SB policies for populations at risk for obesity in clinical/education/social service settings (*k* = 6).

## Discussion

This meta-review provides an overarching synthesis of the evidence for the occurrence of context-related determinants in the implementation of policies promoting a healthy diet and a physically active lifestyle. To the best of our knowledge, this meta-review is the first to focus on theory-based [10] equity factors (e.g., socioeconomic status, cultural, geographical isolation) that might be crucial for the success of health-promoting policies [3].

Across the analyzed documents, the macro-level economic and political context-related determinants were strongly supported as occurring in the implementation of these policies. These determinants might refer to the availability of funds (at the regional or national level) to support sustainable implementation of national/regional policies that are already operating in the setting and might, for example, indirectly support the sustainability of newly implemented policies [41]. Furthermore, political and economic context-related determinants referring to the meso/micro level were also indicated as operating in the implementation process. Such context-related determinants could, for example, refer to the costs of street-scale changes (hindering PA policies) or existing zoning regulations that regulate the use of land in a neighborhood [40]. The third strongly supported context domain encompasses sociocultural determinants at the meso/micro level, representing the beliefs, knowledge, and capabilities of the target groups and policy implementers. Among others, the support obtained for this domain may result from the use of the approaches such as the theoretical domain framework (TDF) [71] in several reviews included in our meta-review. The TDF focuses on identifying determinants referring to individual’s beliefs and includes 20 subcategories within the  domains addressing “beliefs” [71]. In contrast, determinants referring to political or legal system, or geographic characteristics are not captured by the TDF. Thus, the use of the TDF in the included reviews resulted in eliciting multiple implementation determinants related to the beliefs of the target individuals and implementation actors.

We found some differences between context-related determinants indicated as occurring in the implementation processes of healthy diet policies compared with PA/SB policies. The relevance of the meso/micro-level geographical context was stressed for the implementation of PA/SB policies. The characteristics of the built physical environment in a local community in schools or workplaces are among the key correlates of physical activity [33]. Assuming that the implementation of any education, transport-related, or fiscal (e.g., subsidies) policies promoting PA will complement or rely on already existing built PA infrastructure seems plausible. In turn, the legal and political contexts were stressed as important for the implementation of diet policies. The implementation of a new diet policy takes place in a multi-sectorial context involving various governmental agencies (health, safety and hygiene, education, agriculture) and complex industry–government relationships, shaping food production, retail, and catering [20]. That existing regulations (e.g., food labeling and marketing) and commercial stakeholders’ pressure on market-driven solutions [20] will constitute the key context-related determinants for the implementation of diet policies seems likely, whereas these context variables might be of moderate importance for PA/SB policies.

The reviews/stakeholder documents supporting legal context-related determinants often discuss the legal context in close connection to the political context. For example, the documents referred to the fit between the newly implemented policies and other existing policies and regulations or pointed out difficulties navigating existing policies and regulations (e.g. [51, 70, 72]). The implementation of new policies was presented as a process that interacted with existing policies, political pressures, and legal regulations (e.g. [51, 70]).

Ethical determinants of implementation have rarely been addressed, mostly in reference to obesity-related stigma [62]. The stakeholder documents addressing stigma-related issues discussed them in connection to implementers’ beliefs, knowledge, and abilities that disregard stigma and social/environmental contexts in which obesity occurs [62]. According to the health stigma and discrimination framework [73], aspects of stigma refer to the beliefs and capabilities of implementers and the target group. Thus, some ethics-related context determinants might be considered (and reported) as belonging to the same broader category as sociocultural context determinants and not reported directly (e.g., only as discriminatory beliefs). Similarly, the documents that strongly supported the epidemiological context presented this context in conjunction with the cultural appropriateness of the policy implementation [68] or implementers’ beliefs, knowledge, and abilities to recognize the needs of the target population [18]. In summary, the weak support for the ethical domain and the epidemiological determinants might be the result of measurement or analytical strategies in the original research, which resulted in reporting respective determinants as beliefs or capabilities.

Relatively limited support was found in the geographical domain. The geographical context-related determinants often referred to the existing physical (built facilities/fixed equipment) infrastructure to exercise or sell/cater healthy food in settings, such as schools or workplaces, or built facilities in the broader geographical context, such as the infrastructure that allows for active transportation [37]. The relatively limited support for the geographical context might result from the fact that these determinants could be secondary to economic-related context determinants. For example, insufficient funding could lead to difficulties in changing the physical setting of organizations and communities.

The macro-level geographical determinants were preliminarily supported in documents that highlighted geographical isolation, particularly in rural contexts (e.g., healthy food retail, development of sports facilities) [68]. Geographical isolation is rarely addressed in stakeholder documents (see OECD [3] report). Our findings show preliminary support for the geographical context and highlight the importance of recognizing the role of geographical inequalities, which might undermine the implementation of healthy diet and active lifestyle policies.

Policy implementation is a complex, value-driven, decision-making process that occurs in a setting in which multiple values and interests are negotiated toward a shared consensus [74]. Context-related determinants are not static. Because contexts can change, and not all context determinants can be anticipated before policies are implemented, constant policy implementation monitoring is required to adjust implementation and enable the target groups to actively engage with the policy and contribute to its implementation.

Beyond its strengths, the present study has several limitations. The relationships between context-related determinants and other factors operating in the implementation process or their influence on the progress of implementation remain unclear. The data included in reviews/stakeholder documents allow only to conclude which determinants are indicated as present in the process of implementing a healthy diet, PA, and/or SB policies. The coding of context-related determinants relied on the specificity of the operationalization and a description of barriers and facilitators in reviews and stakeholder documents. Thus, several implementation determinants were not assigned to any of the context domains (e.g., time available to implement). The results of the quality evaluation indicated that 48% of reviews and 47% of stakeholder documents presented a low risk of bias attributable to the high quality of the reviewed methods. These findings should inspire a reflection on the insufficient quality of many reviews/stakeholder documents reporting on the determinants of policy implementation. As the CICI framework [10] differentiates between implementation strategies and context, the behaviors of implementers (e.g., staff support for implementation) were not captured as context-related factors. Furthermore, we used the description of the CICI domains as proposed in the original paper [10], which provides relatively general operationalizations illustrated with specific examples. Consequently, the categorization might not capture some of the meso-level determinants (e.g., organizational culture). The original CICI framework captures the political context in a relatively narrow way, and recent research has proposed expanding the framework in terms of the strategic political behavior of representative agents operating within political systems and networks [75]. Several analyzed reviews and stakeholder documents discussed policies (actions involving governments/local authorities) and interventions (actions not involving governments/local authorities [4, 5]). The implementation of interventions might depend more on the sociocultural, economic, and political context-related determinants operating at the meso/micro level (e.g., policies within the organization, implementers’ beliefs, and organizational financial resources available for implementation) relative to the implementation of policies. The included reviews/documents were heterogeneous in terms of the aims of the analyzed policies, their target groups, and settings; therefore, any conclusions should be treated as preliminary. We did not adapt the search string to specific databases and instead we used a broad string fitting various databases. Further research is needed to determine if the use of multiple of narrow strings, adapted to the respective databases, may provide different results and reduce/increase the likelihood of omitting relevant publications. In particular, our strategy to include a long list of keywords referring to the study design (based on [23]) resulted in a high total number of documents identified, which may reduce the likelihood of omitting relevant publications during the first stages of the search process. However, an increased screening workload might also result in an incorrect identification of some entries as irrelevant and thus an incorrect exclusion. The conclusions of any meta-review could be biased if an overlap exists in the original studies analyzed in the included reviews [23]. The heterogeneity of the aims of reviews/documents included in this meta-review reduces the likelihood of such an overlap; yet, some overlap can be expected. The effects of the overlap were not systematically investigated. In line with previous reviews [32, 33], we used a threshold of 60% as indicating strong support and 50% as indicating preliminary support for a context-related determinant. The distinction between these two thresholds is arbitrary, and the patterns of the associations should be confirmed further in a meta-analysis of original research presenting quantitative results. Because a limited number of included reviews (*k* = 7) [19, 39, 46–48, 51, 54] reported quantitative results for any of the context-related determinants, conducting a meta-analysis was not feasible.

## Conclusions

This study provides the first theory-based overarching synthesis of evidence for the support of context-related determinants of the implementation of policies targeting a healthy diet, an increase in physical activity, or a reduction in sedentary behavior. Our findings might alert policymakers, implementers, and researchers to account for social, cultural, economic, and political circumstances when making plans for implementing healthy diet and physical activity policies. Clarifying the role of various context-related domains could improve the understanding of the factors that enable a broad reach and the implementation and sustainability of successful policies.

## Supplementary Information

**Additional file 1: Supplementary Tables S1-S7**.

**Additional file 2.** Details of data extraction.

## Data Availability

All data analysed during this study are either secondary (retrieved from original studies included in the review) or included in this article (i.e., its supplementary information files).

## Abbreviations

PA: Physical activity
SB: Sedentary behaviors
CICI: Context and implementation of complex interventions
