# Supplementary material for: Social, economic, political, and geographical context that counts: meta-review of implementation determinants for policies promoting healthy diet and physical activity
Source: BMC Public Health. 2022 May 26;22:1055. doi: 10.1186/s12889-022-13340-4 (PMC9137101; doi:10.1186/s12889-022-13340-4)
Supplement: Supplementary file 1 — Additional file 1: Supplementary Tables S1-S7. [file 12889_2022_13340_MOESM1_ESM.docx]

**Social, economic, political and geographical context that counts: Meta-review of implementation determinants for policies promoting healthy diet and physical activity**

**Additional File 1**

Table S1

Full list of 5 groups of keywords applied in the searching strategy

| 1. **Implementation: implement*** |
| --- |
| 1. **Determinants, facilitators, and barriers:** barrier* OR facilitat* OR determinant* OR factor* OR affect* OR influenc* OR enhanc* OR improv* OR support* OR gap |
| 1. **The type of action**: polic* OR “prevention program*” OR strateg* OR program* OR practic* OR government*OR law |
| 1. **The design (reviews):** "data synthesis" OR "synthesis of data" OR "descriptive synthesis" OR "evidence synthesis" OR "synthesis of evidence" OR "synthesis of available evidence" OR "best-evidence synthesis" OR "framework synthesis" OR "interpretive synthesis" OR "knowledge synthesis" OR "synthes* of knowledge" OR "literature synthesis" OR "synthesis of literature" OR "synthesis of the literature" OR metasynthesis OR meta-synthesis OR "narrative synthesis" OR "qualitative synthesis" OR "quantitative synthesis" OR "realist synthesis" OR "research synthesis" OR "synthesis of research" OR "synthesized research" OR "review and synthesis" OR "synthesis of qualitative research" OR "thematic synthesis" OR "updated synthesis" OR "congruent synthesis" OR "critical interpretive synthesis" OR "framework synthesis" OR "meta synthesis" OR "qualitative evidence synthesis" OR "refutational synthesis" OR "critical analysis of the literature" OR metaanaly* OR meta-analy* OR "meta analy*" OR "Retrospective analysis" OR "Qualitative analysis" OR "Quantitative analysis" OR "systematic map*" OR "systematic narrative" OR "systematic overview*" OR "systematic review*" OR "systematically review*" OR "systematized review" OR "overview of systematic reviews" OR "systematic assessment" OR "systematic literature mapping" OR "bibliographic search" OR "database search" OR "search of database*"OR "electronic search" OR handsearch* OR "hand search*" OR "search by hand" OR "keyword search" OR "literature search" OR "search of literature" OR "search of the literature" OR "search term*" OR "systematic search" OR "comprehensive search" OR "manual search" OR "article reviews" OR "review of articles" OR "literature review" OR "review literature" OR "review of the literature" OR "overview* of reviews" OR "overview of systematic reviews" OR "qualitative review" OR "quantitative review" OR "realist review" OR "a review" OR "articles are reviewed" OR "authors review" OR "clinical review" OR "complete review" OR "comprehensive review" OR "conduct a review" OR "critical review" OR "critically review" OR "current review" OR "descriptive review" OR "epidemiological review" OR "evidence review" OR "review of the evidence" OR "evidence-based review" OR "integrat* review" OR "research review*" OR "international review" OR "is reviewed" OR "mapping review" OR meta-review OR "narrative review" OR "practitioner review" OR "rapid review" OR "rapid realist* review" OR "review all studies" OR "review and synthesis" OR "review of current evidence" OR "review of current research" OR "review of effectiveness" OR "review of the effectiveness" OR "review of reported cases" OR "review of research" OR "review of reviews" OR "selected for review" OR "selective review" OR "state-of-the-art review*" OR "structured review" OR "theory driven review" OR "scoping study" OR "meta-study" OR "literature-based study" OR "studies were identified" OR "compilation of the literature" OR "compilation of studies" OR "inclusion criteria" OR "exclusion criteria" OR metaregression OR "evidence map*" OR meta-evidence OR "systemic review" |
| 1. **The outcomes:** “physical activity” OR active OR exercise OR sedentary OR sport OR fitness OR sedentary OR “screen use” OR diet OR nutrition OR fat OR snack OR fruit OR vegetable OR fiber OR fibre OR soda OR meal OR food OR “energy intake” OR calorie* OR obes* OR eat* |

*Note*: The keywords were selected based on previous reviews addressing similar issues [1-5]. Applied limiters in published reviews: peer-reviewed publications of reviews applying systematic search of original research (including systematic, scoping, realist reviews, meta-analyses), academic journals, English language only. Applied limiters in stakeholders’ databases: documents approved/published by the respective organization, English language only

References included in the note for Table S1:

1. Hennessy EA, Johnson BT, Keenan C. Best Practice Guidelines and Essential Methodological Steps to Conduct Rigorous and Systematic Meta-Reviews. Appl Psychol Health Well-Being. 2019;11:353–81. https://doi.org/10.1111/aphw.12169

2. Horodyska K, Luszczynska A, Hayes CB, O’Shea MP, Langøien LJ, Roos G, et al. Implementation conditions for diet and physical activity interventions and policies: an umbrella review. BMC Public Health. 2015;15:1250. https://doi.org/10.1186/s12889-015-2585-5

3. Proctor E, Silmere H, Raghavan R, Hovmand P, Aarons G, Bunger A, et al. Outcomes for implementation research: Conceptual distinctions, measurement challenges, and research agenda. Adm Policy Ment Health. 2011;38(2):65–76. https://doi.org/10.1007/s10488-010-0319-7

4. Seward K, Finch M, Yoong SL, Wyse R, Jones J, Grady A, et al. Factors that influence the implementation of dietary guidelines regarding food provision in centre based childcare services: A systematic review. Prev Med. 2017;105:197–205. https://doi.org/10.1016/j.ypmed.2017.09.024

5. Wolfenden L, Jones J, Williams CM, Finch M, Wyse RJ, Kingsland M, et al. Strategies to improve the implementation of healthy eating, physical activity and obesity prevention policies, practices or programmes within childcare services. Cochrane Database Syst. Rev. 2016; 10:CD011779. https://doi.org/10.1002/14651858.cd012439.pub2

Table S2

Additional coding principles for CICI framework

| **Coding principles** |
| --- |
| Determinants were coded as belonging to the micro, meso, and macro level in line with the guidelines and examples provided by Pfadenhauer et al. [1]. |
| **Meso and micro-level determinants**: The implementation determinants domains within the dimensions of CICI framework were assigned to represent a micro -level if they directly referred to individual characteristics; the meso-level, if they referred to organization or community characteristic. Additionally, the micro-level could concern different types of actors involved: delivery system actors, support system actors, target group [2]. |
| In case of coding within **socio-cultural domain** if a determinant was concerning an individual level (the micro-level), however it was not specified whether it relates to the policy target group or implementers/other delivery system actors, then it was coded as concerning both the policy target group and the implementers/other delivery system actors (e.g., “knowledge” or “beliefs” or “work-related stress” of the target group and implementers or actors). |
| In case of **geographical domain**, unless a determinant was not referring to any kind of physical environment or infrastructure, it was not included coded within this domain (e.g., determinants such as “scheduling”, “competing curriculum demands”, “lack of time”, “time available to implement”). Therefore, “lack of space” was coded within the geographical domain, the meso-level, whereas “lack of time” was not included in the coding. Additionally, if a determinant was referring to any kind of physical environment recourses transformed by humans (such as availability of facilities in the school environment) it was coded as infrastructure (the meso-level), whereas determinants referring to broader natural physical environment (such as air quality) were coded as referring to the macro-level within this domain.  Determinants referring to “weather” were not coded within this domain. |
| In case of the physical environment characteristics that were described in a broad manner (e.g., “suitable physical environment”), if a determinant was not explicitly referring to the meso-level (characteristics of specific buildings or facilities relevant for the respective policies, e.g., characteristics of school canteens or sport facilities) then it was coded as belonging to the macro-level of a respective domain. |
| In case of **socio-cultural domain**, if a determinant was concerning behaviors of individuals, it was coded within this domain only if it was related to culture or values or beliefs (e.g., “staff support for physical activity” was not coded within this domain). |
| Every time a determinant was related to any kind of legal issues or regulations it was coded as referring to the **legal context** (the macro-level), e.g., “IACO fits with existing rules/regulations”. In case a determinant was related to governmental taxes, the determinants were coded as belonging to two domains: legal context and economic context (the macro-level). |
| **Strategies for implementation,** as well as elements related directly to implementation (such as implementation climate or readiness for implementation, community readiness, parental support for implementation, formal reinforcement of use of organizational policy plans) were not coded within the context domains, since the CICI framework differentiates between context dimensions, the implementation, and the setting. Context reflects a set of features of circumstances in which the implementation is embedded, whereas implementation refers to planned and deliberately initiated effort with the intention to bring a given intervention/policy into practice within a particular setting [2].  Additionally, if a determinant was only mentioning **characteristics of a policy** that were not immanent for the context and were expressed as a component of p**olicy implementation process or an aspect of policy implementation evaluation**, they were not coded as the implementation determinant fitting a context domain (e.g., “policy is acceptable for user”, “results are observable”, “clear procedures”).  If a determinant’s **name/definition was extremely vague** (e.g., “policy”, without any further explanation or details) and additionally it was not pre-assigned to any category by the authors of the original review, it was not coded as belonging to a context domain. |
| Every time **a determinant did not clearly indicate whom it may concern or it was not clear at which level it is expressed (**micro-, meso- or macro- level), it was not coded (e.g., “physical activity is not culturally accepted”). |
| The presence of “ethical food in stores” was not coded as an implementation determinant referring to the **ethical context**. |

References included in the Table S2:

1. Pfadenhauer LM, Gerhardus A, Mozygemba K, Lysdahl KB, Booth A, Hofmann B, et al. Making sense of complexity in context and implementation: the Context and Implementation of Complex Interventions (CICI) framework. Implemen Sci. 2017;12(1):21. https://doi.org/10.1186/s13012-017-0552-5

2. Leeman J, Birken SA, Powell BJ, Rohweder C, Shea CM. Beyond “implementation strategies”: classifying the full range of strategies used in implementation science and practice. Implemen Sci. 2017;12(1):125. https://doi.org/10.1186/s13012-017-0657-x

Table S3

Barriers and facilitators for implementation: evidence accumulated in reviews summarized according to the CICI framework

| **CICI Contexts** | 1^*^ | 2 | 3 | 4 | 5 | 6 | 7 | 8 | 9 | 10 | 11 | 12 | 13 | 14 | 15 | 16 | 17 | 18 | 19 | 20 | 21 | 22 | 23 | 24 | 25 | **SUM** | **≥ 50% and ≥ 60% reviews^i^** |
| --- | --- | --- | --- | --- | --- | --- | --- | --- | --- | --- | --- | --- | --- | --- | --- | --- | --- | --- | --- | --- | --- | --- | --- | --- | --- | --- | --- |
| **Type of analyzed behavior** | DIET | | | | | | | | PA | | | | | PA, SB | | PA, DIET | | | | | | PA, DIET, OTHER BEHAVIORS | | | |  | |
| **Geographical** | | | | | | | | | | | | | | | | | | | | | | | | | | | |
| Broader physical environment (e.g., geographical isolation) (macro-level) |  |  | + |  |  | + |  | + |  | + | + |  |  |  | + |  | + |  | + |  |  | + |  |  |  | **9** |  |
| Infrastructure in the setting (meso/micro level) |  |  | **+** |  |  | **+** |  | **+** | **+** | **+** | **+** |  | **+** | **+** |  | **+** | **+** | **+** | **+** |  |  | + |  |  | + | **14** | **✓** |
| **Epidemiological** | | | | | | | | | | | | | | | | | | | | | | | | | | | |
| Distribution of diseases, disease burden, demographics(macro-level) |  |  |  | **+** |  | **+** |  |  |  |  |  |  |  |  |  |  |  |  |  |  |  |  |  |  |  | **2** |  |
| Needs of target groups (psychosocial, physical) (meso/micro level) | + |  | **+** |  |  | **+** |  |  |  |  |  |  | **+** |  |  |  |  |  | **+** |  |  | + | + |  | + | **8** |  |
| **Socio-cultural** | | | | | | | | | | | | | | | | | | | | | | | | | | | |
| Culture-related ideas, symbols, roles, values (macro-level) | + | **+** |  | **+** |  | **+** |  |  | **+** | **+** |  |  |  |  |  | **+** |  |  | **+** |  |  | + | + |  |  | **10** |  |
| Target group: knowledge, beliefs, abilities (meso/micro level) | + |  | **+** | **+** | **+** | **+** |  | **+** | **+** | **+** | **+** | **+** | **+** | **+** | **+** |  | **+** |  | **+** |  |  | + | + | + |  | **18** | **✓✓** |
| Implementers: knowledge, beliefs, abilities (meso/micro level) | + | **+** | **+** |  | **+** | **+** | **+** | **+** | **+** |  | **+** | **+** | **+** | **+** | **+** | **+** |  | **+** | **+** |  |  | + | + |  | + | **19** | **✓✓** |
| **Economic** | | | | | | | | | | | | | | | | | | | | | | | | | | | |
| Economic resources of communities (macro-level) | + | **+** | **+** | **+** | **+** | **+** |  | **+** |  | **+** | **+** | **+** |  |  |  | **+** |  |  | **+** |  |  |  | + |  | + | **14** | **✓** |
| Individual’s or organization’s economic resources (meso/micro-level) | + | **+** |  |  | **+** | **+** | **+** | **+** | **+** | **+** |  |  |  | **+** | **+** | **+** | **+** |  | **+** | **+** |  | + |  | + | + | **17** | **✓✓** |
| **Ethics** | | | | | | | | | | | | | | | | | | | | | | | | | | | |
| Standards of conduct, ethical norms, stigma(macro-level) |  |  |  |  |  |  |  |  |  |  |  |  |  |  |  |  |  |  |  |  |  |  |  |  |  | **0** |  |
| Target group: Individual’s belief - standards, norms, stigma (meso/micro level) |  |  |  |  |  |  |  |  |  |  |  |  |  |  |  |  |  |  |  |  |  |  |  |  |  | **0** |  |
| Implementers: Individual’s belief - standards, norms, stigma (meso/micro level) |  |  |  |  |  |  |  |  |  |  |  |  |  |  |  |  |  |  |  |  |  |  |  |  |  | **0** |  |
| **Political** | | | | | | | | | | | | | | | | | | | | | | | | | | | |
| Interrelated policies, political pressure (macro-level) | + | **+** | **+** | **+** | **+** | **+** |  | **+** | **+** |  | **+** |  | **+** | **+** | **+** | **+** |  |  | **+** |  |  |  | + |  | + | **16** | **✓✓** |
| Policies in organizations involved/partner organizations (meso/microlevel) |  | **+** | **+** | **+** | **+** | **+** | **+** | **+** |  |  | **+** | **+** |  | **+** | **+** | **+** |  | **+** | **+** |  |  |  | + |  | + | **16** | **✓✓** |
| Sectorial policies: health care, education, food production and retail system properties (macro-level) | + | **+** | **+** | **+** | **+** | **+** |  | **+** |  |  | **+** | **+** |  | **+** |  |  |  |  | **+** |  |  |  | + |  |  | **12** |  |
| **Legal** | | | | | | | | | | | | | | | | | | | | | | | | | | | |
| Rules/regulations established to protect population rights and societal interests; enforced laws (macro-level) | + | **+** | **+** | **+** | **+** | **+** |  |  |  |  | **+** |  |  |  |  |  |  |  | **+** |  |  |  |  |  | + | **9** |  |

*Note*: PA - physical activity; SB - sedentary behavior. ^*^Numbers of the reviews (1-25) reflect the numbers of the reviews in the Supplement 1 (see also the list below). + - the reviews/stakeholder documents, that provided an explicit reference for a significant role/importance of a respective context-related implementation determinant. The context domain may be divided into macro- (nation-wide), meso- (organizational)/ micro- (individual) levels.

^i^ ≥ 50% (≥13 reviews) indicating preliminary support for determinant is marked with 🗸; ≥ 60% (≥15 reviews) indicating strong support for determinant is marked with 🗸🗸

Reference list of reviews (1-25) included in the Table S3

1. Seward K, Finch M, Yoong SL, Wyse R, Jones J, Grady A, et al. Factors that influence the implementation of dietary guidelines regarding food provision in centre based childcare services: A systematic review. Prev Med. 2017;105:197–205. https://doi.org/10.1016/j.ypmed.2017.09.024

2. Cullerton K, Donnet T, Lee A, Gallegos D. Playing the policy game: a review of the barriers to and enablers of nutrition policy change. Public Health Nutr. 2016;19:2643–53. https://doi.org/10.1017/S1368980016000677

3. Middel CNH, Schuitmaker-Warnaar TJ, Mackenbach JD, Broerse JEW. Systematic review: a systems innovation perspective on barriers and facilitators for the implementation of healthy food-store interventions. Int J Behav Nutr Phys Act. 2019;16:108. https://doi.org/10.1186/s12966-019-0867-5

4. Bergallo P, Castagnari V, Fernández A, Mejía R. Regulatory initiatives to reduce sugar-sweetened beverages (SSBs) in Latin America. PLoS One. 2018;13:e0205694. https://doi.org/10.1371/journal.pone.0205694

5. Eykelenboom M, van Stralen MM, Olthof MR, Schoonmade LJ, Steenhuis IHM, Renders CM. Political and public acceptability of a sugar-sweetened beverages tax: a mixed-method systematic review and meta-analysis. Int J Behav Nutr Phys Act. 2019;16:1356. https://doi.org/10.1186/s12966-019-0843-0

6. Houghtaling B, Serrano EL, Kraak VI, Harden SM, Davis GC, Misyak SA. A systematic review of factors that influence food store owner and manager decision making and ability or willingness to use choice architecture and marketing mix strategies to encourage healthy consumer purchases in the United States, 2005–2017. Int J Behav Nutr Phys Act. 2019;16:5. https://doi.org/10.1186/s12966-019-0767-8

7. Wang D, Stewart D. The implementation and effectiveness of school-based nutrition promotion programmes using a health-promoting schools approach: a systematic review. Public Health Nutr. 2013;16:1082-100.  https://doi.org/10.1017/S1368980012003497

8. McIsaac J-LD, Spencer R, Chiasson K, Kontak J, Kirk SFL. Factors Influencing the Implementation of Nutrition Policies in Schools: A Scoping Review. Health Educ Behav. 2019;46:224–50. https://doi.org/10.1177/1090198118796891

9. Nathan N, Elton B, Babic M, McCarthy N, Sutherland R, Presseau J, et al. Barriers and facilitators to the implementation of physical activity policies in schools: A systematic review. Prev Med. 2018;107:45–53. https://doi.org/10.1016/j.ypmed.2017.11.012.

10. Rees R, Kavanagh J, Harden A, Shepherd J, Brunton G, Oliver S, Oakley A. Young people and physical activity: a systematic review matching their views to effective interventions. Health Educ Res. 2006;21:806-25. https://doi.org/10.1093/her/cyl120

11. Heath GW, Brownson RC, Kruger J, Miles R, Powell KE, Ramsey LT. Task Force on Community Preventive Services. The Effectiveness of Urban Design and Land Use and Transport Policies and Practices to Increase Physical Activity: A Systematic Review. J Phys Act Health. 2006;3:S55-S76. https://doi.org/10.1123/jpah.3.s1.s55

12. Leone L, Pesce C. From Delivery to Adoption of Physical Activity Guidelines: Realist Synthesis. Int J Environ Res Public Health. 2017;14:1193. https://doi.org/10.3390/ijerph14101193

13. Weatherson KA, Gainforth HL, Jung ME. A theoretical analysis of the barriers and facilitators to the implementation of school-based physical activity policies in Canada: a mixed methods scoping review. Implement Sci. 2017;12:36. https://doi.org/10.1186/s13012-017-0570-3

14. Cassar S, Salmon J, Timperio A, Naylor P-J, van Nassau F, Contardo Ayala AM, et al. Adoption, implementation and sustainability of school-based physical activity and sedentary behaviour interventions in real-world settings: a systematic review. Int J Behav Nutr Phys Act. 2019;16:120. https://doi.org/10.1186/s12966-019-0876-4

15. Umstattd Meyer MR, Perry CK, Sumrall JC, Patterson MS, Walsh SM, Clendennen SC, et al. Physical Activity-Related Policy and Environmental Strategies to Prevent Obesity in Rural Communities: A Systematic Review of the Literature, 2002-2013. Prev Chronic Dis. 2016;13:E03. https://doi.org/10.5888/pcd13.150406

16. Naylor P-J, Nettlefold L, Race D, Hoy C, Ashe MC, Wharf Higgins J, McKay HA. Implementation of school based physical activity interventions: A systematic review. J Prev Med. 2015;72:95-115. https://doi.org/10.1016/j.ypmed.2014.12.034

17. Dugdill L, Brettle A, Hulme C, Bartys S, Long A. A review of effectiveness of workplace health promotion interventions on physical activity and what works in motivating and changing employees health behaviour. Project Report. London, UK.: NICE. 2007. http://www.nice.org.uk/guidance/index.jsp?action=download&o=36393

18. Wolfenden L, Barnes C, Jones J, Finch M, Wyse RJ, Kingsland M, et al. Strategies to improve the implementation of healthy eating, physical activity and obesity prevention policies, practices or programmes within childcare services. Cochrane database Syst Rev. 2020;2:CD011779. https://doi.org/10.1002/14651858.cd011779.pub2

19. van der Kleij R, Coster N, Verbiest M, van Assema P, Paulussen T, Reis R, Crone M. Implementation of intersectoral community approaches targeting childhood obesity: a systematic review. Obes Rev. 2015;16:454-72. https://doi.org/10.1111/obr.12273

20. McFadyen T, Chai LK, Wyse R, Kingsland M, Yoong SL, Clinton-McHarg T, et al. Strategies to improve the implementation of policies, practices or programmes in sporting organisations targeting poor diet, physical inactivity, obesity, risky alcohol use or tobacco use: a systematic review. BMJ Open. 2018;8:e019151. https://doi.org/10.1136/bmjopen-2017-019151

21. Anderson LM, Quinn TA, Glanz K, Ramirez G, Kahwati LC, Johnson DB, et al. The effectiveness of worksite nutrition and physical activity interventions for controlling employee overweight and obesity: a systematic review. Am J Prev Med. 2009;37:340-57. https://doi.org/10.1016/j.amepre.2009.07.003

22. Wierenga D, Engbers LH, van Empelen P, Duijts S, Hildebrandt VH, van Mechelen W. What is actually measured in process evaluations for worksite health promotion programs: a systematic review. BMC public health. 2013;13:1190. https://doi.org/10.1186/1471-2458-13-1190

23. Durlak JA, DuPre EP. Implementation matters: a review of research on the influence of implementation on program outcomes and the factors affecting implementation. Am J Community Psychol. 2008;41:327-50. https://doi.org/10.1007/s10464-008-9165-0

24. Wolfenden L, Regan T, Williams CM, Wiggers J, Kingsland M, Milat A, et al. Strategies to improve the implementation of workplace-based policies or practices targeting tobacco, alcohol, diet, physical activity and obesity. Cochrane Database Syst Rev. 2016;34:175. https://doi.org/10.1002/14651858.cd012439.pub2

25. Herlitz L, MacIntyre H, Osborn T, Bonell C. The sustainability of public health interventions in schools: a systematic review. Implement Sci. 2020;15:4. https://doi.org/10.1186/s13012-019-0961-8

Table S4

Barriers and facilitators for implementation: evidence accumulated in stakeholder documents summarized according to the CICI framework

| **CICI Contexts** | 1s^*^ | 3s | 3s | 4s | 5s | 6s | 7s | 8s | 9s | 10s | 11s | 12s | 13s | 14s | 15s | 16s | 17s | **SUM** | | | | | **≥50% and ≥60% of documents^i^** |
| --- | --- | --- | --- | --- | --- | --- | --- | --- | --- | --- | --- | --- | --- | --- | --- | --- | --- | --- | --- | --- | --- | --- | --- |
| **Type of analyzed behavior** | DIET | | | | PA | | DIET, PA, SB AND OTHER BEHAVIORS | | | | | | | | | | |  | | | | | |
| **Geographical** |  | | | |  | |  | | | | | | | | | | |  | | | | | |
| Broader physical environment (e.g., geographical isolation) (macro-level) | + |  |  | + | + |  | + | + |  |  |  | + | + | + |  | + |  | **9** | | | | **✓** | |
| Infrastructure in the setting  (meso/micro level) | + |  |  |  | **+** |  | **+** | **+** |  | **+** |  | **+** | **+** | **+** |  |  |  | **8** | | | |  | |
| **Epidemiological** | | | | | | | | | | | | | | | | | | | | | | | |
| Distribution of diseases, disease burden, demographics  (macro-level) |  | **+** | **+** | **+** |  |  | **+** |  |  | **+** | **+** | **+** | **+** | **+** | **+** | **+** |  | **11** | | | | **✓✓** | |
| Needs of target groups (psychosocial, physical)  (meso/micro level) | + |  |  |  |  |  | **+** |  |  | **+** | **+** | **+** | **+** | **+** |  | **+** |  | **8** | | | |  | |
| **Socio-cultural** | | | | | | | | | | | | | | | | | | | | | | | |
| Culture-related ideas, symbols, roles, values  (macro-level) | + |  | **+** | **+** |  |  | **+** | **+** | **+** | **+** | **+** | **+** | **+** | **+** | **+** | **+** |  | **13** | | | **✓✓** | | |
| Target group: knowledge, beliefs, abilities  (meso/micro level) | + |  | **+** | **+** |  | **+** | **+** | **+** | **+** | **+** | **+** |  |  |  |  | **+** | **+** | **11** | | | **✓✓** | | |
| Implementers: knowledge, beliefs, abilities  (meso/micro level) | + |  |  | **+** |  | **+** | **+** | **+** | **+** | **+** | **+** |  | **+** | **+** |  | **+** | **+** | **12** | | | **✓✓** | | |
| **Economic** | | | | | | | | | | | | | | | | | | | | | | | |
| Economic resources of communities  (macro-level) | + | **+** | **+** | **+** |  |  | **+** | **+** | **+** | **+** | **+** | **+** | **+** | **+** | **+** | **+** |  | **14** | | | **✓✓** | | |
| Individual’s or organization’s economic resources  (meso/micro-level) | + |  | **+** | **+** | **+** |  | **+** | **+** |  | **+** | **+** | **+** | **+** | **+** | **+** | **+** |  | **13** | | | **✓✓** | | |
| **Ethics** | | | | | | | | | | | | | | | | | | | | | | | |
| Standards of conduct, ethical norms, stigma  (macro-level) |  |  |  |  |  |  |  | **+** |  |  |  |  |  | **+** |  | **+** |  | **3** | |  | | | |
| Target group: Individual’s belief - standards, norms, stigma (meso/micro level) |  |  |  |  |  |  |  |  |  |  |  |  |  |  |  |  |  |  | |  | | | |
| Implementers: Individual’s belief - standards, norms, stigma (meso/micro level) |  |  |  |  |  |  |  |  |  |  |  |  |  |  |  |  |  |  | |  | | | |
| **Political** | | | | | | | | | | | | | | | | | | | | | | | |
| Interrelated policies, political pressure  (macro-level) | + | **+** | **+** | **+** |  |  | **+** | **+** | **+** | **+** | **+** | **+** | **+** | **+** | **+** | **+** |  | **14** | | **✓✓** | | | |
| Policies in organizations involved/partner organizations (meso/microlevel) | + | **+** |  | **+** |  | **+** | **+** | **+** | **+** | **+** |  | **+** | **+** | **+** | **+** | **+** |  | **13** | | **✓✓** | | | |
| Sectorial policies: health care, education, food production and retail system properties (macro-level) |  | **+** |  | **+** |  |  | **+** | **+** | **+** | **+** | **+** | **+** | **+** | **+** | **+** | **+** |  | **12** | | **✓✓** | | | |
| **Legal** | | | | | | | | | | | | | | | | | | | | | | | |
| Rules/regulations established to protect population rights and societal interests; enforced laws (macro-level) | + |  | **+** | **+** |  |  |  | **+** | **+** | **+** |  |  | **+** | **+** | **+** | **+** |  | **10** | **✓** | | | | |

*Note*: PA - physical activity; SB - sedentary behavior. ^*^Numbers of the stakeholder documents (1s-17s) reflect the numbers of the documents in the Supplement 1 (see also the list below). + - the reviews/stakeholder documents, that provided an explicit reference for a significant role/importance of respective context-related implementation determinants. The context domain may be divided into macro- (nation-wide), meso- (organizational)/micro- (individual) levels;

^i^ ≥ 50% (≥ 9 stakeholder documents) indicating preliminary support for determinant is marked with 🗸; ≥ 60% (≥ 10 stakeholder documents) indicating strong support for determinant is marked with 🗸🗸

Reference list of stakeholder documents (1s-17s) included in the Table S4

1. National Health and Medical Research Council. Australian dietary guidelines. Canberra, A.C.T.: National Health and Medical Research Council; 2013.

2. European Commission. Inspiring the shift from nutrition policy to implementation: How existing data can support nutrition decision-making in Guatemala. Brussels: Directorate General International Cooperation and Development; 2019. http://www.nipn-nutrition-platforms.org/IMG/pdf/nipn_guatemala_case_study_-_brief_-_july_2019.pdf. Accessed 8 Oct 2020.

3. Adler NE, Cutler DM, Fielding JE, Galea S, Glymour MM, Koh HK, et al. Addressing Social Determinants of Health and Health Disparities: A Vital Direction for Health and Health Care. NAM Perspectives. 2016;6. . https://doi.org/10.31478/201609t

4. Heymsfield S, Aronne LJ, Eneli I, Kumar R, Michalsky M, Walker E, et al. Clinical Perspectives on Obesity Treatment: Challenges, Gaps, and Promising Opportunities. NAM Perspectives. 2018;8. https://doi.org/10.31478/201809b

5. National Institute for Health and Care Excellence (NICE). Physical activity in the workplace: Public health guideline [PH13]. UK: National Institute for Health and Care Excellence (NICE); 2008. https://www.nice.org.uk/guidance/ph13/resources/physical-activity-in-the-workplace-pdf-1996174861765. Accessed 8 Oct 2020

6. National Institute for Health and Care Excellence (NICE). Physical activity: walking and cycling: Public health guideline [PH41]. UK: National Institute for Health and Care Excellence (NICE); 2012. https://www.nice.org.uk/guidance/ph41/resources/physical-activity-walking-and-cycling-pdf-1996352901061. Accessed 8 Oct 2020.

7. National Health and Medical Research Council. Clinical practice guidelines for the management of overweight and obesity in adults, adolescents and children in Australia. Melbourne: National Health and Medical Research Council; 2013.

8. National Health and Medical Research Council. Cultural competency in health: A guide for policy, partnerships, and participation. Canberra, A.C.T.: National Health and Medical Research Council; 2006.

9. National Health and Medical Research Council. Infant feeding guidelines: Information for health workers. Canberra, A.C.T.: National Health and Medical Research Council; 2012.

10. Colagiuri S, Johnson G. Case for Action proposal: A Comprehensive Type 2 Diabetes Prevention Program. Canberra, A.C.T.: Submitted by the NHMRC Research Translation Faculty Diabetes Mellitus Steering Group; 2014.

11.Teede H, Harrison C, editors. Case for Action proposal: Obesity prevention through preventing excess weight gain during pregnancy and postpartum. Canberra, A.C.T.: Submitted by the NHMRC Research Translation Faculty Obesity Steering Group; 2014.

12. European Commission. Reviews of scientific evidence and policies on nutrition and physical activity. Objective area A2: effectiveness and efficiency of policies and interventions on diet and physical activity. Brussels: Publications Office of the European Union; 2018. https://data.europa.eu/doi/10.2875/337727. (2018). Accessed 12 Oct 2020.

13. Dietz WH, Brownson RC, Douglas CE, Dreyzehner JJ, Goetzel RZ, Gortmaker SL, et al. Chronic Disease Prevention: Tobacco, Physical Activity, and Nutrition for a Healthy Start: A Vital Direction for Health and Health Care. NAM Perspectives. 2016;6. https://doi.org/10.31478/201609j

14. Dietz WH, Belay B, Bradley D, Kahan S, Muth ND, Sanchez E, et al. A Model Framework That Integrates Community and Clinical Systems for the Prevention and Management of Obesity and Other Chronic Diseases. NAM Perspectives. 2017;7. https://doi.org/10.31478/201701b

15. Zellner S, Bowdish L. The ROI of Health and Well-Being: Business Investment in Healthier Communities. NAM Perspectives. 2017;17. https://nam.edu/wp-content/uploads/2017/11/The-ROI-of-Health-and-Well-Being.pdf. Accessed 28 Oct 2020

16. National Institute for Health and Care Excellence (NICE). Obesity: working with local communities: Public health guideline [PH42]. UK: National Institute for Health and Care Excellence (NICE); 2012. https://www.nice.org.uk/guidance/ph42. Accessed 28 Oct 2020.

17. National Institute for Health and Care Excellence (NICE). Obesity prevention: Clinical guideline [CG43]. UK: National Institute for Health and Care Excellence (NICE); 2006. https://www.nice.org.uk/guidance/cg43. Accessed 28 Oct 2020.

Table S5

Description of analyzed material and principles of data coding according to the CICI framework

|  | **Reviews** | **Stakeholder documents** |
| --- | --- | --- |
| Number of included documents | 25 | 17 |
| Total number of original studies | 747 | Not available |
| Type of action | both policies and interventions | both policies and interventions |
| Analyzed behaviors | 1. dietary behaviors only (*k* = 8; 32%), 2. physical activity only (*k* = 5; 20%) 3. both, physical activity and sedentary behaviors (*k* = 2; 8%); 4. diet behaviors and physical activity *(k* = 6, 24%); 5. diet behaviors, physical activity, and other behaviors (*k* = 4; 16% [e.g., smoking cessations, alcohol use, drug use, oral health, relaxation at work, various social and mental health issues such as violence or bullying, eating disorders or well-being]). | 1. diet behaviors only (*k* = 4; 23%); 2. physical activity only (*k* = 2; 12%) 3. diet, physical activity, sedentary behaviors and other behaviors (*k* = 11; 65%) |
| Populations analyzed | 1. general populations of adults (*k* = 10; 40%), 2. employees (*k* = 4; 16%) 3. children and adolescents (*k* = 11; 44%). | 1. general population (*k* = 7, 41.2%;); 2. clinical populations with various types of illnesses (e.g., various types of clinical populations combined together, e.g., children with obesity, adolescents or adults combined with other population at high risk of developing diabetes combined, people with various chronic diseases, e.g., heart disease, diabetes) (*k* = 2, 11.8%); 3. specific communities (e.g., local communities (*k* = 2, 11.82%); 4. population of pregnant and postpartum women at risk for obesity (*k* = 2, 11.8%); 5. populations including only people with overweight or obesity (children, adolescents, and adults) (*k* = 2, 11.8%); 6. employees (*k* = 1; 5.9%), 7. vulnerable population (e.g., children of low-income families, pregnant women, older adults with disabilities and age related illness, prisoners (*k* = 1; 5.9%). |
| Policy implementation settings | 1. school settings (*k* = 10, 40%) 2. mixed settings (*k* = 6; 24%); 3. workplace settings (*k* = 4; 16%); 4. food retail (e.g., food stores, food serving outlets) (*k* = 2; 8%); 5. rural community settings; urban planning and housing environment and transportation (*k* = 1; 4%); 6. various physical activity facilities in the community (*k* = 1; 4%); | 1. mixed settings (*k* = 9; 53%); 2. communities or community and health care settings (*k* = 3; 17.7%); 3. clinical practice settings (*k* = 2; 11.8%); 4. health, social services, educational, and other settings involved in prevention of overweight among pregnant and postpartum women and educating on infant feeding (*k* = 2; 11.8%); 5. workplace settings only (*k* = 1; 5.9%); |

| Table S6  Examples of context-related barriers/facilitators within domains of the CICI framework | |
| --- | --- |
| **Context domains**  **Context-related determinants based on the CICI framework** | **Examples of context-related determinants**  **(TYPE OF ADDRESSED BEHAVIOR BY THE REVIEW/STAKEHOLDER DOCUMENT)*** |
| **Geographical** | |
| **Broader physical environment**  **(macro-level)** | - Product supply challenges in remote areas (geographic isolation) (DIET) [3]; - Store type or location (e.g., small store retailers noted fewer product deliveries, unavailability of products, and a higher expense for healthy options in rural areas) (DIET) [6]; - School location and rurality (e.g., smaller, rural schools have greater challenges with accessing nutritional foods and organizational supports; larger and more urban schools emerged as more likely to have resources in place) (DIET) [8]; - Physical barriers to cycling to work including state and lack of cycle paths, weather, pollution, and cycle locking facilities (PA/DIET) [17]; - Suitable physical environment/resources available - ‘the absence of a suitable physical environment’ (e.g., the limited availability of healthy foods in stores) (PA/DIET) [19] |
| **Infrastructure in the setting**  **(meso/micro-level)** | - Limited or lacking infrastructure (due to lack of space or equipment, time and/or cost barriers) in retailer ability to offer healthy food and beverages (DIET) [6]; - Appropriate school infrastructure (e.g., cafeterias, gardens, and compost and recycling infrastructure, were reported to facilitate implementation) (DIET) [8]; - Inappropriate or inadequate facilities in schools (e.g., gym kit, showers) or lack of facilities for leaving bicycles at school (PA/SB) [10]; - Reducing the convenience of lifts at the workplace; improving the stair environment and décor at the workplace, more changing and showering facilities (PA/DIET) [17]; - Adapting the built environment to promote physical activity on community level (e.g., changing urban environments to ensure a safe environment for exercise) (PA/DIET) [12s] |
| **Epidemiological** | |
| **Disease distribution, disease burden, demographics**  **(macro-level)** | - Scope of age-protection identified (e.g., when implementing regulations to advertising or other forms of promotion of unhealthy food and/or drinks) (DIET) [4]; - Use of screening within high-risk populations in prevention programs (participation in a prevention program requires the exclusion of existing incidences of, e.g., diabetes) (PA/DIET) [10s]; - Consideration of SES, ethnic, gender differences and how to reach all groups/impact of an intervention on different groups, particularly high-risk groups (PA/DIET) [12s]; - Consideration in particular people who require tailored information and support, especially inactive, vulnerable groups by local authorities and partners (PA/DIET) [16s] |
| **Target group needs**  **(meso/micro-level)** | - Consumer demands and demographics (e.g., some retailers perceived their consumer base to lack knowledge of healthy diets, and to be disinterested in improving dietary behaviors to benefit health; however, seniors and consumers with noncommunicable diseases were thought to be more willing to purchase healthy products) (DIET) [6]; - Role of a food store in promoting consumer health including supporting families within the consumer base, children’s health outcomes, and helping to mitigate high observed rates of noncommunicable diseases (retailers in one study perceived store changes to impact the health of the community, however in another study retailers worried that promoting consumer health might be considered offensive to their base) (DIET) [6]; - Perceived relevance of the approach (e.g., targeting childhood obesity) for the target population [19]; - Access to specific target populations - ensuring culturally appropriate and tailored programs for high-risk populations (e.g., to offer people at high risk but without diabetes a tailored prevention intervention depending on the state of their health, their location, their work commitments, their socio‐economic status, and their preference) (PA/DIET) [10s] |
| **Socio-cultural** | |
| **Culture-related ideas, symbols, roles, values**  **(macro-level)** | - Similar socio-cultural backgrounds of retailers and intervention/research personnel perceived to be beneficial for establishing partnerships (DIET) [6]; - Negative stereotypes about young women’s abilities in sport (PA/SB) [10]; - Parental constraint (due to safety concerns, monitoring of leisure time, particular cultural values) (PA/SB) [10]; - Changes culturally acceptable for users (PA/DIET) [19]; - Taking account of cultural or religious values, for example, the need for separate physical activity sessions for men and women, or in relation to body image, or beliefs and practices about hospitality and food (PA/DIET) [16s] |
| **Target group: knowledge, beliefs, abilities**  **(meso/micro-level)** | - Retailers’ notion that healthy/produce products are often perceived by consumers to be more expensive to purchase and have less convenience attributes when compared to less healthy foods and beverages (DIET) [6]; - Consumers’ tastes were perceived to favor unhealthy foods and beverages rather than healthy products (DIET) [6]; - Outcome beliefs, feasibility, and perceived importance of participants [14] AND Perceived enthusiasm of adolescents about the intervention (PA/SB) [14]; - Lack of confidence and competence, feelings of discomfort and self-consciousness about bodies (young women only), lack of motivation and ‘inertia’, preference for other activities, and lack of knowledge about the benefits of physical activity (PA/SB) [10]; - Changes are considered relevant/suitable for the target population (PA/DIET) [19] |
| **Implementers: knowledge, beliefs, abilities**  **(meso/micro-level**) | - Staff perceptions of what foods children liked or disliked) [1]; ‘Knowledge’ (e.g., staff have limited general nutrition knowledge and poor knowledge of the sector menu dietary guidelines) [1]; ‘Beliefs about capabilities (e.g., food service staff lack confidence in their kitchen math skills and cooking skills) [1], AND 'Skills' (e.g., highly trained and skilled staff) (DIET) [1]; - ‘Social influences’ (e.g., staff communicating and collaborating; well established social networks to share information; networking and knowledge brokering - information seeking/sharing through social networks) (DIET) [1]; - Coordination with other agencies (e.g., involvement and support of experts in sports, health, and education) and communication (e.g., clear/short communication between teachers, sections, and teams) (PA/SB) [14]; - High self-efficacy to implement new approach [19] AND The availability of skills and knowledge among professionals to implement new approach (PA/DIET) [19]; - Implementers' lack of recognition of cultural beliefs, cultural appropriateness, and cultural values in planning and delivering the health promotion programs/policies (PA/DIET) [8s] |
| **Economic** | |
| **Economic resources of communities**  **(macro-level)** | - Financial resources: food costs - higher cost of healthy foods (impact of rising food costs on very limited budgets) (DIET) [1]; - Prioritizing of economic prosperity as the main barrier to policy change (demonstrated by a lack of support for regulatory intervention, with the justification that it could interfere with market-driven economies) (DIET) [2]; - (Financial) political support (financial resources made available for implementation) (PA/DIET) [19]; - Funding and material resources - the availability of funding (materials and space for sustaining an intervention) (PA/DIET) [25] |
| **Individual/organizational economic resources**  **(meso/micro-level)** | - Limited resources, money, and time of health advocates as significant barriers to policy change (e.g., the volunteer advocates felt themselves to be at a distinct disadvantage against paid, full-time lobbyists) (DIET) [2]; - Compared with state- and nationally focused initiatives, local initiatives able to leverage funding and support through different, often less competitive routes (DIET) [2]; - Business model favoring quick-grab items rather than grocery products (business models described as dependent on profits and the convenience of operations) (DIET) [6]; - Making activities more affordable for young people (PA/SB) [10]; - Lack of consistent funding for the initiative over long-term (PA/SB) [15] |
| **Ethics** | |
| **Standards of conduct, ethical norms, stigma**  **(macro-level)** | - Health and consumer rights as a basis to establish responsibility for cultural competency and set out legislative and ethical obligations for individuals and organization (PA/DIET) [8s]; - Changes in the design of the clinical delivery systems required to develop a standard of care that responds with sensitivity to the bias and stigma that accompany obesity (most health professionals remain relatively unaware of the relationship of obesity to social factors such as stigma and bias0 (PA/DIET) [14s]; - Taking account of cultural or religious values (e.g., the need for separate physical activity sessions for men and women, or in relation to body image, or beliefs and practices about hospitality and food; they also take account of religious and cultural practices) (PA/DIET/OTHER BEHAVIORS) [16s] |
| **Target group: standards, norms, stigma**  **(meso/micro-level)** | not found |
| **Target group: standards, norms, stigma**  **(meso/micro-level)** | not found |
| **Political** | |
| **Interrelated policies, political pressure**  **(macro-level)** | - Prioritisation of government policies that resulted in short-term economic benefit, over policies related to longer-term health outcomes (DIET) [2] - Conflicted decision-making. (e .g., competing agendas which prioritised certain nutrition issues over others, for example, food safety over food security in health department or when the government department responsible for protecting agricultural producers was the same department advising the public about dietary intake) (DIET) [2] - Pressure from industry, e.g., ‘intense lobbying' (industry had a lot more points of interaction with government than health organizations as providers of tax revenue, major employers, through international linkages, and as holders of specialised knowledge) (DIET) [2] - Ensure solutions are politically palatable. For a solution to be politically palatable it needed to align with at least one goal of the government as well as the prevailing political ideology (DIET) [2] - Social-political context, e.g., political support for the initiative, the initiative fits with existing rules/regulations (PA/DIET) [19] |
| **Policies in organizations involved/partner organizations**  **(meso/micro-level)** | - Retailers incomplete control over the foods and beverages available in food stores (e.g., if the store was a chain or corporate location, stocking decisions were determined within upper management) (DIET) [6]; - Clear hierarchical structures within organizations (PA/SB) [14] - Clear protocols, tasks, and agreements among organizations (PA/SB) [14]; - Formal reinforcement of new strategies use in organization policy/plans (PA/DIET) [19]; - Integration of new programming - refers to the extent to which an organization can incorporate innovation into its existing practices and routines (PA/DIET) [23] |
| **Sectorial policies: health care, education, food production and retail system properties**  **(macro-level)** | - Government silos (coordinating various departments to work together can be challenging as they tend to work in ‘silos’ and prioritise their own objectives, e.g., some government departments can take a narrow perspective of their responsibilities, for example: local government identifying its sole role in food policy as the regulation of food hygiene and safety) (DIET) [2]; - Contract agreements dictated unhealthy product stocking, promotions, and placement in prime consumer areas (although good for business, have negative potential impacts on consumer health) (DIET) [6]; - Supplier product purchasing policies (i.e., purchasing amount, package sizes, return policies) (DIET) [6]; - Non-synergic relationship, within national PA plans regarding the goals of the sport, education, and urban planning sectors (PA/SB) [12] |
| **Legal** | |
| **Rules/regulations established to protect population rights/societal interests, enforced laws**  **(macro-level)** | - Tension created by proposing legislation as a solution within nations with a liberal tradition of personal responsibility, individual choice, and free markets (e.g., a change in the government resulting in food and nutrition policies being watered down or rescinded on the basis of the revised policy being more industry-friendly, or because of personal responsibility arguments) (DIET) [2]; - Governmental legislation as a potential facilitator for the implementation of interventions (DIET) [3]; - Lack of strong official (national) implementation strategy (DIET) [3]; - Zoning regulations that preclude mixed-use neighborhoods (in terms of community-scale and street-scale urban design and land use policies and practices) (PA/SB) [11]; - Regional or national health policies could support sustainability by legitimising health promotion in schools (PA/DIET) [25] |
| *Note:* * - each example is followed by a reference number according to the list of references inserted under Table S4 and S5 in current Additional File 1 | |

| Table S7  Specific populations and settings targeting diet, physical activity (PA) and sedentary behaviors (SB) | | | | | | |
| --- | --- | --- | --- | --- | --- | --- |
| **Behaviors analyzed in the studies included in the review/stakeholders documents (diet or PA or SB)** | **Specific target population/policy implementation setting** | | | | | |
|  | **Children/adolescents at school setting** | | **Employees at workplace setting** | | **Children/adolescents/adults at risk for obesity**  **(in clinical, education, or social services settings) including:**  **(a) population of pregnant women or pregnant/postpartum women at risk for obesity**  **(b) clinical population (at high risk of developing diabetes)**  **(c) overweight and obese children, adolescents, adults** | |
|  | **Reviews**  **[references]** | **Stakeholder**  **documents**  **[references]** | **Reviews**  **[references]** | **Stakeholder documents**  **[references]** | **Reviews**  **[references]** | **Stakeholder documents**  **[references]** |
| **Diet** | *k=* 3 [1, 2, 3] | - | - | - | - | *k=* 1 [16] |
| **Diet/PA/SB** | *k* = 3 [4, 5, 6] | - | *k* = 4 [11, 12, 13, 14] | - | - | *k=* 5 [17, 18, 19, 20, 21] |
| **PA** | *k* = 3 [7, 8, 9] | - |  | *k* = 1 [15] | - |  |
| **PA/SB** | *k* = 1 [10] | - | - | - | - |  |
| **Number of documents** | 44% (10 out of 25) | - | 16% (4 out of 25) | 5,9% (1 out of 17) | - | 35% (6 out of 17) |
| **TOTAL NUMBER** | **23.8% (10 out of 42)** | | **11.9% (5 out of 42)** | | **14.3% (6 out of 42)** | |

Reference list of reviews (1-21) included in the Table S7

1. Seward K, Finch M, Yoong SL, Wyse R, Jones J, Grady A, et al. Factors that influence the implementation of dietary guidelines regarding food provision in centre based childcare services: A systematic review. Prev Med. 2017;105:197–205. https://doi.org/10.1016/j.ypmed.2017.09.024

2. McIsaac J-LD, Spencer R, Chiasson K, Kontak J, Kirk SFL. Factors Influencing the Implementation of Nutrition Policies in Schools: A Scoping Review. Health Educ Behav. 2019;46:224–50. https://doi.org/10.1177/1090198118796891.

3. Wang D, Stewart D. The implementation and effectiveness of school-based nutrition promotion programmes using a health-promoting schools approach: a systematic review. Public Health Nutr. 2013;16:1082–100. https://doi.org/10.1017/S1368980012003497.

4. Herlitz L, MacIntyre H, Osborn T, Bonell C. The sustainability of public health interventions in schools: a systematic review. Implement Sci. 2020;15:4. https://doi.org/10.1186/s13012-019-0961-8.

5. Naylor P-J, Nettlefold L, Race D, Hoy C, Ashe MC, Wharf Higgins J, et al. Implementation of school based physical activity interventions: A systematic review. Prev Med. 2015;72:95–115. https://doi.org/10.1016/j.ypmed.2014.12.034.

6. Wolfenden L, Barnes C, Jones J, Finch M, Wyse RJ, Kingsland M, et al. Strategies to improve the implementation of healthy eating, physical activity and obesity prevention policies, practices or programmes within childcare services. Cochrane database of Syst Rev. 2020;2:CD011779. https://doi.org/10.1002/14651858.cd011779.pub2.

7. Nathan N, Elton B, Babic M, McCarthy N, Sutherland R, Presseau J, et al. Barriers and facilitators to the implementation of physical activity policies in schools: A systematic review. Prev Med. 2018;107:45–53. https://doi.org/10.1016/j.ypmed.2017.11.012.

8. Rees R, Kavanagh J, Harden A, Shepherd J, Brunton G, Oliver S, et al. Young people and physical activity: a systematic review matching their views to effective interventions. Health Educ Res. 2006;21:806–25. https://doi.org/10.1093/her/cyl120.

9. Weatherson KA, Gainforth HL, Jung ME. A theoretical analysis of the barriers and facilitators to the implementation of school-based physical activity policies in Canada: a mixed methods scoping review. Implement Sci. 2017;12:36. https://doi.org/10.1186/s13012-017-0570-3.

10. Cassar S, Salmon J, Timperio A, Naylor P-J, van Nassau F, Contardo Ayala AM, et al. Adoption, implementation and sustainability of school-based physical activity and sedentary behaviour interventions in real-world settings: a systematic review. Int J Behav Nutr Phys Act. 2019;16:219. https://doi.org/10.1186/s12966-019-0876-4.

11. Wierenga D, Engbers LH, van Empelen P, Duijts S, Hildebrandt VH, van Mechelen W. What is actually measured in process evaluations for worksite health promotion programs: a systematic review. BMC public health. 2013;13:1190. https://doi.org/10.1186/1471-2458-13-1190

12. Wolfenden L, Regan T, Williams CM, Wiggers J, Kingsland M, Milat A, et al. Strategies to improve the implementation of workplace-based policies or practices targeting tobacco, alcohol, diet, physical activity and obesity. Cochrane Database Syst. Rev. 2016;34:175. https://doi.org/10.1002/14651858.cd012439.pub2.

13. Anderson LM, Quinn TA, Glanz K, Ramirez G, Kahwati LC, Johnson DB, et al. The effectiveness of worksite nutrition and physical activity interventions for controlling employee overweight and obesity: a systematic review. Am J Prev. Med. 2009;37:340–357. https://doi.org/10.1016/j.amepre.2009.07.003.

14. Dugdill L, Brettle A, Hulme C, Bartys S, Long A. A review of effectiveness of workplace health promotion interventions on physical activity and what works in motivating and changing employees health behaviour. Project Report. London, UK: NICE. 2007. http://www.nice.org.uk/guidance/index.jsp?action=download&o=36393. Accessed 8 Oct 2020.

15. National Institute for Health and Care Excellence (NICE). Physical activity in the workplace: Public health guideline [PH13]. UK: NICE; 2008. https://www.nice.org.uk/guidance/ph13/resources/physical-activity-in-the-workplace-pdf-1996174861765. Accessed 8 Oct 2020

16. Heymsfield S, Aronne LJ, Eneli I, Kumar R, Michalsky M, Walker E, et al. Clinical Perspectives on Obesity Treatment: Challenges, Gaps, and Promising Opportunities. NAM Perspectives. 2018;8. https://doi.org/10.31478/201809b.

17. Teede HJ, Harrison CL, Lombard CB, Boyle J, East C, Brown W. Case for Action proposal: Obesity prevention through preventing excess weight gain during pregnancy and postpartum.: Submitted by the NHMRC Research Translation Faculty Obesity Steering Group. 2014. https://research.monash.edu/en/publications/case-for-action-proposal-obesity-prevention-through-preventing-ex. Accessed 12 Oct 2020.

18. Colagiuri S, Johnson G. Case for Action proposal: A Comprehensive Type 2 Diabetes Prevention Program. Canberra, A.C.T.: Submitted by the NHMRC Research Translation Faculty Diabetes Mellitus Steering Group; 2014. https://www.nhmrc.gov.au/about-us/publications/cases-action. Accessed 8 Oct 2020.

19. Dietz WH, Belay B, Bradley D, Kahan S, Muth ND, Sanchez E, et al. A Model Framework That Integrates Community and Clinical Systems for the Prevention and Management of Obesity and Other Chronic Diseases. NAM Perspectives, 2017;7. https://doi.org/10.31478/201701b.

20. National Health and Medical Research Council. Infant feeding guidelines: Information for health workers. Canberra, A.C.T.: National Health and Medical Research Council; 2012. https://www.nhmrc.gov.au/about-us/publications/infant-feeding-guidelines-information-health-workers. Accessed 12 Oct 2020.

21. National Health and Medical Research Council. Clinical practice guidelines for the management of overweight and obesity in adults, adolescents and children in Australia. Melbourne: NHMRC; 2013. https://www.nhmrc.gov.au/about-us/publications/clinical-practice-guidelines-management-overweight-and-obesity. Accessed 8 Oct 2020
